# Bio-optimized complex valued spatiotemporal GNN for herbal species classification

**DOI:** 10.1038/s41598-026-41760-4

**Published:** 2026-04-01

**Authors:** Prashant Vats, Shailender Vats, Avani Sharma, Saneh Lata Yadav, Deevesh Chaudhary

**Affiliations:** 1 Department of Computer Science and Engineering, Manipal University Jaipur, Dehmi Kalan, Jaipur, Rajasthan 303007 India; 2 Department of Computer Application, Accurate Institute of Management and Technology, Greater Noida, Uttar Pradesh 201310 India; 3 Department of Information Technology, Manipal University Jaipur, Dehmi Kalan, Jaipur, Rajasthan 303007 India; 4 Department of Computer Science and Engineering, K. R. Mangalam University, Sohna Rural, Gurgaon, Haryana 122103 India; 5 Department of Data Science and Engineering, Manipal University Jaipur, Dehmi Kalan, Jaipur, Rajasthan 303007 India

**Keywords:** Herb classification, Graph neural network, Leaf morphology, Sustainable agriculture, Graph convolutional neural network, Botanical features, Hierarchical manta ray foraging optimization, Computational biology and bioinformatics, Mathematics and computing, Plant sciences

## Abstract

Herbs have long been integral to various health and medicinal benefits. Identifying the correct herb species from thousands of diverse options is a laborious and time-consuming process. To address this issue, an automated computer vision system that reduces the traditional labor-intensive work of herbal species classification is needed. In this paper, we propose a novel method for automated herb classification via a complex-valued spatiotemporal graph convolutional neural network (AHC-CVSTGCN) with hierarchical manta ray foraging optimization. The objective of our research work is to develop a robust, effective, and optimized classification model driven by graph neural networks on a diverse array of morphological features of various herbal leaves. Input images are collected from FLAVIA and the medical leaf dataset. The images are first preprocessed utilizing Multiple Local Particle Filter (MLPF) to remove background noise and enhance quality, and then Revised Tunable Q-Factor Wavelet Transform (RQFWT) extracts relevant features such as shape, color, and texture. Finally, CVSTGCN classifies the images, with HMRFO optimization applied to further improve accuracy. Our proposed approach bridges the gaps in the classification of various herbal species, empowering medicine practitioners to make informed decisions. Experimental evaluation demonstrated that our approach significantly outperforms existing methods by achieving 99.40% high accuracy, 99.11% high precision, and 99.12% high recall. By contributing a reliable and effective solution for automated herbal species classification, this work presents a crucial paradigm shift for medicinal plant science and health care practitioners.

## Introduction

The classification of plants or herbal species is a rapidly evolving field within computer vision, gaining attention because of its relevance in biodiversity preservation, agriculture, medicine, and ecological studies^1^. Plants and their species are indispensable parts of the ecosystem, contributing significantly to human and animal life^2^. They serve as sources of food, medicine, oxygen, shelter, and aesthetic value, often exhibiting multifunctional roles across categories^3^. The immense diversity of flora, with approximately 374,000 known plant species, 308,312 of which are vascular, reflects both the richness and complexity of nature^4^. Among these, a significant portion serve for medicinal purposes, highlighting the need for precise identification^5^. In a world where digitization continues to transform various domains, the emergence of digital plant collections such as virtual herbaria has opened new pathways for managing and accessing botanical information^6^. These digital repositories consist of extensive multimedia data, like textual descriptions, images, and audio-visual content related to plant species. However, the challenge lies in efficiently retrieving and interpreting this vast volume of information, especially when the visual characteristics of different plant species can be strikingly similar^1,7^. In this context, plant and herbal species recognition through computer vision presents a significant leap forward^8^. The morphological features of plants, especially the color, texture, and shape of leaves, play a central role in species identification. Among various plant parts, leaves are particularly useful due to their relatively stable and distinctive morphological attributes^9,10^. Once matured, a leaf retains a specific shape, texture, color, and vein structure, making it a reliable source of identification^11^. These visible traits carry inherent patterns that reflect both intra-class consistency and inter-class diversity^12^. Leaf texture, in particular, holds rich and quantifiable information that distinguishes one species from another^13^. Texture can be described as the visual surface pattern of an object that results from an image’s pixel intensities’ spatial distribution^14,15^. It is a fundamental visual attribute that captures characteristics such as roughness, smoothness, regularity, and directionality^16^. While the human visual system can intuitively sense these patterns, the ability to define, interpret, and analyze them computationally requires sophisticated mathematical modeling. Texture in images may arise from natural processes or be artificially introduced, yet it consistently forms the foundation for identifying subtle distinctions in visual data. Recognizing plant species through computer vision remains a complex task due to natural variability in leaf appearance, environmental factors, and overlapping visual traits among species. In addition, limitations related to dataset diversity, feature extraction, and optimization strategies continue to affect classification performance. Recent studies have explored advanced bio-inspired and trend-aware metaheuristic optimization strategies, such as the Parrot Optimizer and trend-aware mechanisms, to improve convergence stability and learning effectiveness in health-related and neural network–based classification tasks^17^^18^.

To overcome this limitation, a computer vision system approach for classification of a diverse array of herbal species is proposed. It uses multiple local particle filters (MLPF) as a preprocessing step that enhances tracking and robustness by modeling the local variations, thus helping in capturing detailed structural differences in herbal leaves. Furthermore, Revised Tunable Q-Factor Wavelet Transform (RQFWT) for feature extraction offers superior time-frequency localization, making it suitable for extracting discriminative features from textured leaf surfaces. This transform adapts better to non-stationary signals compared to traditional wavelets. Finally, the extracted morphological features are fed to complex-valued spatio-temporal graph convolutional neural network (CVSTGCN) for the classification of herbs, followed by hierarchical manta ray foraging optimization (HMRFO) to provide a powerful global optimization strategy by mimicking the intelligent foraging behavior of manta rays. The optimization is improving classification performance while avoiding local optima. These advanced techniques offer high accuracy, robustness, and better generalization capabilities in complex and noisy herbal image datasets compared to conventional classifiers.

The main contributions of this research work are summarized below:

- Multiple local particle filter (MLPF) is effective in eliminating background noise at the same time maintaining a critical point of the leaf, overcoming the problem of poor generalization and overlapping leaves of the past.
- The revised tunable Q-factor wavelet transform (RQFWT) is able to simultaneously extract shape, texture, and color features to overcome the problems of texture-only or shape-only methods
- Complex value spatio-temporal graph convolutional neural network (CVSTGCN) models intricate spatial and temporal cravings, and include additional discriminative and informative characteristics than customary CNN or LSTM-based models.
- Hierarchical manta ray foraging optimization (HMRMO) is an efficient network optimization approach that eliminates overfitting and enhances classification accuracy and ROC performance.
- The hybrid system outperforms on various measurements, like accuracy, recall, F1-score, precision, specificity, FPR, FNR, and ROC, thus being applicable in large-scale automated classification of herbs.
- The technique is able to beat the difficulties of earlier methods, including sensitivity to a noisy background, overlapping leaves, fine morphological variations, and the impossibility of measuring shape, color, and texture simultaneously.
- The proposed system is a robust and trustworthy solution, which can be used in real-life conditions to identify herbal species in an accurate and efficient manner.

By using morphological features of leaves as discriminative and applying graph- based convolutional neural network optimized through a bio- inspired algorithm, we are able to achieve substantial gains in accuracy and other parameters. The proposed approach is effective under varied conditions and provides accurate herb species classification. The remaining paper is arranged as follows: Section “Literature review” provides the literature survey, Section “Proposed methodology” discusses the proposed methodology, the results are discussed in Section “Results and discussion”, followed by the conclusion in Section “Conclusion”.

## Literature review

Research in the classification of herb and plant species has gained the attention of a substantial number of researchers in the fields of herbal medicine, plant taxonomy, and horticulture. Traditional methods of herb classification depend upon manual intervention, such as by smell or touch, and handcrafted features such as leaf structure. But these features often require a professional to analyze the various features and identify the specific herb. Additionally, handcrafted features are often affected by various conditions such as heat and cold exposure, rain, light conditions, etc. With the emergence of computer vision techniques, specifically convolutional neural networks, the classification task is much easier and more accurate. The learning capability of CNN converged with preprocessing techniques makes it standard for image-based classification models. Also, it minimizes the need for manual feature engineering. Most of the deep learning-based herb classifiers consider the shape and texture of the leaf, but are not robust and accurate enough to handle other complex morphological features of leaf structure. Various works suggested in the literature, according to deep learning-driven automated herb classification systems, are surveyed here.

### Traditional machine learning approaches

In 2022 Twum et al.^19^ have presented a textural analysis to identify medicinal plants using log Gabor filters. The approach was tested on a dataset created by Ghana’s Centre of Plant Medicine Research, which included forty- nine plant species and standards from the Flavia and Swedish Leaf datasets. When tested against nine supervised classifiers, the Log gabor filter beat the gabor filters that were extensively employed in the field. In 2022, Kaur et al.^20^ suggested herbal plant modelling and classification using a random forest classifier that con- siders various features. The process of feature extraction extracts features, like leaf area, perimeter, length, width, leaf area covered by a rectangle, leaf percentage in the rectangle, and leaf pixels in four distinct quadrants. The random forest classifier was used to classify various species of herbal plants using shape as the primary characteristic. In 2023 Islam et al.^21^ have presented classifying medicinal plants with a particle swarm optimized cascaded network. The suggested technique extracted features from a pre-trained ResNet50 model using a cascaded architecture. Following feature optimization through Particle Swarm Optimization (PSO), the plants were categorized using a Support Vector Machine (SVM).

### Deep learning approaches

In 2020, Muneer et al.^22^ presented an effective and automated classification technique to identify Malaysian herbs used in cooking and medicine. A mobile app related to the classifier to provide real-time classification after many classifiers were examined to create an efficient classifier in the suggested system. The suggested system made use of two classifiers: Support Vector Machine (SVM) and Deep Learning Neural Network (DLNN). In 2022, Ariful Hassan et al.^23^ have presented deep learning for the detection of medicinal plants from leaf images. One of the most important characteristics for identifying the plant was its leaf. There weren’t many standard leaf data sets for medicinal plants, though. Since deep learning models have demonstrated high recognition accuracy, the work covers both the creation of a standard data set and the employment of deep learning methods to identify plants from their leaves. In 2022 Azadnia, et al.^24^ have presented a reliable and universal approach for classifying various plants that makes use of the spatial attention and channel attention modules. The dataset included 900 verified photos of three distinct plant classes: oregano, toxic, and weed. Deep learning networks were made more efficient by the attention mechanisms, which enable them to precisely focus on all pertinent input pieces.

### Hybrid and optimization-based approaches

In 2023, Kumar, et al.^25^ have presented multi-layered perceptron and machine learning technique for the categorization of leaf images of herbal plants. To use these herbs as medication, it was essential to recognize them. The issue of identification needs to be resolved for the public to use herbs, and practical, affordable ways should be suggested. Any plant can be recognized by images of its leaves. Ghosh et al.^26^ have suggested that the particle swarm optimization algorithm’s functionality to evolve the YOLO model’s near-optimal architecture for effective weed classification. During the data preprocessing stage, we used image segmentation and augmentation techniques to improve weed classification. We sourced data from two different origins, namely ICAR-DWR and TNAU. However, this approach was limited by its dependency on manually curated datasets and may not generalize well to environments with high variability in weed species, lighting conditions affecting classification robustness. Dhal et al.^27^ have suggested MOODM-Net, a Multi-Objective Optimization (MOO)-based deep network for DM prediction. The most informative features from various data sources were found using a novel hybrid feature selection (FS) method that combines multi-objective Harris Hawk Optimization (HHO) with Gray Wolf Optimization (GWO). An essential component of data fusion in smart healthcare, the FS step seeks to glean valuable insights from potentially redundant and noisy data gathered from various sources, including genetic data, wearable sensors, and Electronic Health Records (EHRs).The summary of the literature review is shown in Table 1.

**Table 1:** Summary of literature survey.

Authors(s)	Technique	Key findings	Advantages	Disadvantages
Twum et al.^19^	Log Gabor + ML	Texture-based plant ID	Simple, effective	Fails with overlapping
Kaur et al.^20^	Random Forest	Shape-based leaf classification	Captures shape well	High computation, limited feature relations
Islam et al.^21^	PSO + ResNet50 + SVM	Optimized feature classification	Better accuracy	Dataset-dependent, sensitive to complex scenes
Muneer et al.^22^	DLNN + SVM	Mobile herb classifier	Real time, automated	Limited testing under varied conditions
Ariful Hassan et al.^23^	CNN	Leaf-based recognition	High accuracy	Small dataset, sensitive to environment
Azadnia et al.^24^	CNN	Limited real-world applicability	Efficient, precise	Limited real-world applicability
Kumar et al.^25^	Multi-layer Perceptron (MLP)	Practical leaf classification	Simple, practical	Underperform in complex scenes
Ghosh et al.^26^	PSO + YOLO	Optimized weed detection	Improved detection	Manual dataset dependency, limited generalization
Dhal et al.^27^	MOODM-Net	Optimized DM prediction	Improved accuracy	High computational cost

### Research gap and motivation

The generic overview of the recent literature demonstrates that automated classification of herbal species belongs to a significant task in botany, traditional medicine, and computer vision systems, the correct identification of plants can aid in medical research, biodiversity conservation and intelligent agriculture. The existing application performance in this field is still underdeveloped and the current approaches to the field are usually sensitive to noisy backgrounds, overlapping leaves, fine differences in the shape of leaves and color differences which decrease the accuracy and reliability. A powerful and extensive herb classification is a difficult undertaking in automated identification of plants based on images, as there is high inter-class similarity, intra-class variation, and environmental influences (occlusion or change in light). Different technologies have been employed by different researchers in the literature to tackle this problem with the use of different technologies being used which include DLNN, KNN and RF-based deep learning models. Under AHC-ST-DLNN, the technique possesses the problem of poor generalization and absence of total dependency on locality of features of leaves. The TA-MPI-LGF-KNN method has the issue of using texture-based features and thus performing badly in the case of overlapping or broken leaves. The MHPDF-RF algorithm is more accurate but still has a high cost of computation and it has a low ability to establish complicated relationships between features. These technologies cannot be used to simultaneously capture shape, color, and texture characteristics along with spatial-temporal dependencies with optimal model parameters, which is essential to effective herb classification in field environments. In literature, very few approaches have been presented that integrate noise reduction, advanced feature extraction, complex graph-based learning, and parameter optimization into a unified framework to solve this problem; these drawbacks and challenges motivated the development of the current research work.

The proposed AHC-CVSTGCN method directly addresses several limitations of existing herbal classification approaches by integrating multiple complementary techniques. It removes background noise and preserves critical leaf details through the use of Multiple Local Particle Filter (MLPF), effectively addressing issues related to poor generalization and overlapping leaves. At the same time, it extracts shape, texture, and color features simultaneously using the Revised Tunable Q-Factor Wavelet Transform (RQFWT), overcoming the limitations of approaches that rely solely on texture or shape. The method further models complex spatial and temporal dependencies via the Complex Value Spatio-Temporal Graph Convolutional Neural Network (CVSTGCN), providing richer and more discriminative feature representations than traditional CNN or LSTM-based models. Additionally, the network parameters are optimized efficiently using Hierarchical Manta Ray Foraging Optimization (HMRFO), which reduces overfitting and enhances both accuracy and ROC performance. Collectively, these innovations resolve key challenges faced by previous methods, resulting in a highly accurate, robust, and reliable automated herbal classification framework that is suitable for large-scale deployment.

## Proposed methodology

**Fig. 1:**
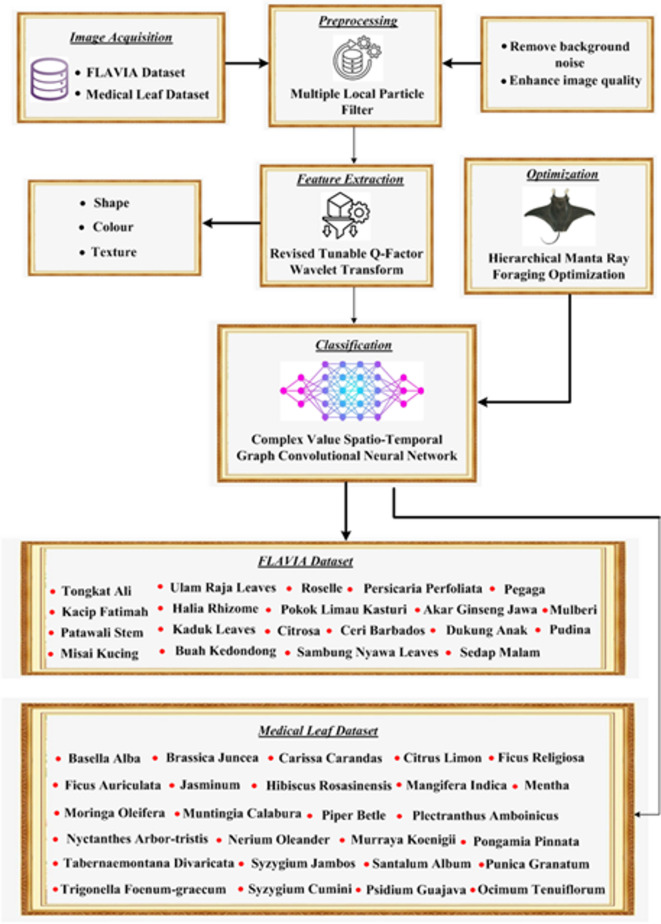
Block diagram of proposed AHC-CVSTGCN.

In this section, an optimized neural network for Automated Herb Classification (AHC-CVSTGCN) is proposed. First, preprocessing using Multiple Local Particle Filter (MLPF) is applied to the FLAVIA dataset to improve image quality and minimize noise. Revised Tunable Q-Factor Wavelet Transform (RQFWT) is used to extract features related to the color, texture, and shape of leaves. Then, using the Hierarchical Manta Ray Foraging Optimization (HMRFO) optimization technique, the CVSTGCN is applied for herb classification. Herbs are categorized into 20 varieties, such as Kacip Fatimah, Tongkat Ali, and others. The efficacy of the AHC-CVSTGCN approach is demonstrated by its significantly high accuracy, precision, and recall when implemented in Python and compared to other methods such as textural analysis for medicinal plant identification using log gabor filters (TA-MPI-LGF-KNN)^21^, an effective and automated method for classifying herbs based on shape and texture features using deep learning (AHC-ST-DLNN)^22^, and a random forest classifier employed for modeling and classification of herbal plants considering various features utilizing machine learning (MHPDF-RF)^20^. The block diagram of the proposed AHC-CVSTGCN approach is illustrated in Fig. 1.

The comprehensive explanation about each step is provided below.

### Objective formulation

The objective of this research is to design an effective herbal species classification framework that extracts discriminative shape, texture, and color features using RQFWT and performs accurate classification through the CVSTGCN network, while ensuring robustness against noise and inter-class similarity. This objective is mathematically expressed as the optimization of classification performance in Eq. (1).

1 $$\begin{aligned} \max _{\Theta } A = f\!\left( \Phi _{\text {RQFWT}}(I),\, \Theta _{\text {CVSTGCN}} \right) \end{aligned}$$

where, *I* denotes the input herb leaf image, $\Phi _{\text {RQFWT}}$ represents the RQFWT-based feature extraction process, $\Theta _{\text {CVSTGCN}}$ denotes the trainable parameters of the CVSTGCN model, and *A* indicates the classification accuracy. The objective focuses on maximizing the discriminative capability and generalization performance compared to existing methods.

### Image acquisition

High-quality leaf images were collected from benchmark datasets to ensure reliable feature extraction and model evaluation, as follows:

#### FLAVIA dataset

**Fig. 2:**
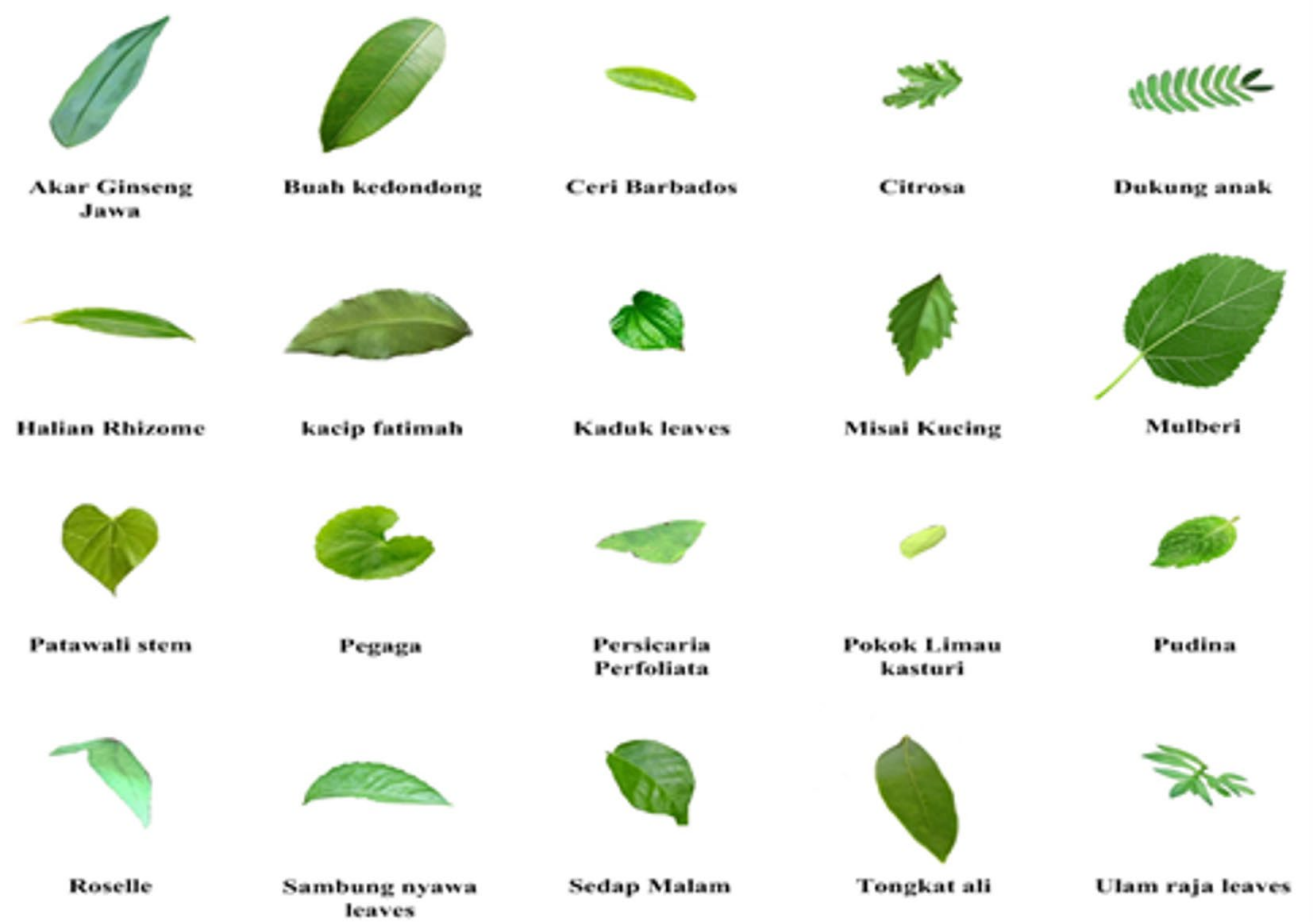
Input sample of FLAVIA dataset.

Initially, the input images from the FLAVIA dataset^28^. The Flavia Dataset, curated by Gaurav Neupane, is a comprehensive collection of 1907–1908 grayscale images representing 32 distinct leaf species, captured under controlled conditions to support machine learning applications in plant classification. Each image, sized 128 ×  128 pixels, displays the leaf’s upper surface against a uniform background, with the leaf blade isolated and the petiole excluded. Using scanners and digital cameras, the photos were taken of common plants in China’s Yangtze Delta region, specifically from Nanjing University and the Sun Yat-Sen Arboretum. Between 50 and 77 photos were taken of each species. This dataset is highly valuable for tasks and feature extraction, pattern recognition, and deep learning, providing a rich resource for practitioners and researchers in computer vision and botany aiming to develop automated plant species identification systems. The input samples are given in Fig. 2.

In addition to dataset composition, the FLAVIA dataset exhibits clear inter-class and intra-class variations. Inter-class variations arise from inherent morphological differences among plant species, including leaf shape, size, margin characteristics, vein structure, texture, and surface appearance, which are essential for species discrimination but increase classification complexity due to partial visual similarities among certain species. Simultaneously, intra-class variations exist within the same species due to differences in leaf maturity, orientation, scale, illumination conditions, background effects, and natural biological diversity. Environmental factors such as lighting changes, shadows, and minor deformations further contribute to this variability. These variations make the dataset challenging and realistic, providing a robust benchmark for evaluating the proposed model under real-world conditions.

#### Medical leaf dataset

The Medical Leaf Dataset has been collected in different places and the images have been taken using a mobile phone or a scanner^29^. The initial data was 2515 images of 1600 ×  1200 pixels with forty different classes. The DeepHerb dataset, which is publicly accessible, has been reduced to 1835 photos in 30 of the 40 classes that were initially provided. No additional cleaning or processing was necessary because the data was already in a standard format that was cleaned and met the study’s requirements. In the current study, it was used in its purest form. The herb species that are part of the current dataset have the following scientific names: Amaranthus viridis, Artocarpus, and Alpinia Galanga Ficus Auriculata, Ficus Religiosa, Hibiscus Rosasinensis, Mentha, Moringa Oleifera, Muntingia Calabura, Murraya Koenigii, Nerium Oleander, Nyctanthes Arbor-tristis, Ocimum Tenuiflorum, Piper Betle, Plectranthus, Azadirachta Indica, Basella Alba, Brassica Juncea, Carissa Carandas, Citrus Limon, Citrus Limon Syzygium Cumini, Syzygium Jambos, Tabernaemontana Divaricata, Santalum Album, Amboinicus, Pongamia Pinnata, Psidium Guajava, Punica Granatum, and Trigonella Foenum-graecum.

### Pre-processing using multiple local particle filter (MLPF)

The preprocessing step using MLPF^30^ is discussed in this section. MLPF is employed to remove background noise and enhance image quality. The MLPF was selected for preprocessing due to its strong ability to handle complex, noisy backgrounds common in herb leaf images. Unlike traditional filters, MLPF processes multiple local regions using a probabilistic approach, preserving fine details and enhancing image clarity. Its localized adaptability makes it ideal for retaining key shape and texture features, improving the quality of input for accurate herb classification. The images are divided into small regions for localized filtering, as denoted in Eq. (2).

2 $$\begin{aligned} q (y_{1} \mid x_{1-l}) = \prod _{i=1}^{c} q(y_{i}^{j} \mid x_{1-l-1}, x_{l}^{i}) \end{aligned}$$

where, $y_{l}\mid x_{1-l}$ denotes local region identification, $y_{l}^{j}$ denotes the various features of the image, $x_{l}^{i}$ denotes the outliers, and $x_{1-l-1}$denotes the neighbor data in filter layer. Multiple particles are generated within each local region. In the MLPF, multiple candidate particles within each local region represent hypotheses for the true pixel values. Weighting these particles based on neighborhood similarity allows effective detection and suppression of outliers. By removing these outliers, the filter ensures that only reliable, noise-free pixel information is preserved. This significantly benefits subsequent feature extraction using RQFWT, enabling accurate capture of morphological patterns such as leaf vein directionality and texture details without interference from noise or corrupted pixels. Each particle repre- sents a candidate pixel value hypothesis, sampled from the neighborhood distribution as expressed in Eq. (3).

3 $$\begin{aligned} q(y_{l}^{j} \mid x_{1-l-1}, x_{l}^{i}) = \int q (y_{l}^{j}, h_{l}^{j} \mid x_{1-l-1}, x_{l}^{i}) \, e^{h_{l}^{j}} \, dh_{l}^{j} \end{aligned}$$

where, $h_{j}^{i}$ denotes the particle generation and e denotes the random variable. A weight is assigned to each particle based on its similarity to neighboring pixel intensities, as expressed in Eq. (4).

4 $$\begin{aligned} q(y_{0:l}^{j} \mid x_{1-l-1}, x_{l}^{i}) \approx \sum _{j=1}^{M} v^{(i,j)} \, \vartheta \left( y_{0:l}^{i} - y_{0:l}^{(i,j)}\right) \end{aligned}$$

where $y_{0:l}^{i}$ and $y_{0:l}^{(i,j)}$ denote the inaccurate data, $v^{(i,j)}$ represent data flaws, and $\vartheta (\cdot )$ are the operations used to detect and minimize measurement errors. Particles are combined using their weights to compute the final denoised pixel value, as expressed in Eq. (5).

5 $$\begin{aligned} q(y_{0:l}^{j} \mid x_{1-l-1}, x_{l}^{i}) = q(y_{l}^{i} \mid y_{l-1}^{i}) \, q(y_{0:l-1}^{i} \mid y_{0:l-2}, y_{l-1}^{i}) \times p(y_{l}^{i} \mid y_{l}^{i}, \hat{h}_{l}^{i}) \end{aligned}$$

where, $y_{0:l-2}$ and $y_{l-1}^{i}$ denotes the weighted particle fusion and$\hat{h}_{l}^{i}$ represents subspace in the filter. The original pixels at each location are replaced with the estimated values, and this process is repeated across the image to reconstruct the filtered, enhanced image, as expressed in Eq. (6).

6 $$\begin{aligned} v_{l}^{(i,j)} \propto \tilde{v}_{l-1}^{(i,j)} \, q(x_{l}^{i} \mid y_{l}^{i}, \hat{h}_{l}^{i}) \end{aligned}$$

where, $\tilde{v}_{l-1}^{(i,j)}$ denotes the removed outliers and $\alpha$ denotes the image reconstruction. Finally, to quantitatively capture the noise reduction effect, the enhanced local variance of filtered pixels is computed as Eq. (7).

7 $$\begin{aligned} \sigma = \frac{1}{N} \sum _{i=1}^{N} (y_i - \bar{y})^{2} \cdot w_i \end{aligned}$$

where, $\sigma$ represents the variance of the denoised pixel values, $\bar{y}$ is the local mean, $w_i$ denotes the particle weights, and *N* is the number of particles in the local region. This demonstrates that the MLPF adaptively suppresses noise while preserving important structural features. Subsequently, the RQFWT is employed to extract features from the preprocessed images.

### Feature extraction using revised tunable Q-factor wavelet transform (RQFWT)

In this section, RQFWT^31^ is employed to extract the morphological features from images, such as shape, texture, and color features. The RQFWT was chosen for its ability to extract detailed texture, shape, and color features with high time-frequency resolution. Unlike traditional filters, RQFWT offers adjustable Q-factors for better feature localization, making it more effective in capturing subtle differences in herb leaf images. Its revised design also improves noise resistance and computational efficiency, making it ideal for accurate herb classification. A set of wavelet filters was applied based on Q-factor-controlled frequency ranges, as expressed in Eq. (8).

8 $$\begin{aligned} \frac{\partial \omega _{d}^{j}(P, x)}{\partial P} = \frac{\left[ (x + 2i - 2)P + x - 2\right] \left[ (P + 1)x - 2\right] ^{i - 2} \, d_{R} \pi }{(P + 1)^{i + 1} \, x^{i - 1}} \end{aligned}$$

where the $\partial \omega _{d}^{j}$ is increasing the transform function, $P$ the bandwidth has a zero point, x decreases the function, $i$ the center frequency is controlled, $d_{R}$ is designed to meet the criteria and $\pi$ data function. Fine localization in both time and frequency domains was achieved, as expressed in Eq. (9).

9 $$\begin{aligned} R_j^t = \frac{|a^t(j)|}{\sum _{i=1}^{M^T} |a^t(i)|} \end{aligned}$$

where, $R_j^t$ denotes the sub-band, $a^t$length of sub band coefficient, $j$ represents the sampling point, $i$ are constituted to transform bank and $M^T$ are the bandwidth of the filter. The image was decomposed into multiple layers of detail and approximation coefficients, as expressed in Eq. (10)

10 $$\begin{aligned} \tilde{Z} = \{ G(\tilde{B}_j),\ j = |\tilde{B}| \} \end{aligned}$$

Here, $|\tilde{B}|$ the cardinal number of the set $\tilde{B}_j$, $G$ defines the common characteristic subspaces and $j$ represents the sampling point. Texture orientation and directionality in leaf patterns were captured by applying directional filters within the RQFWT sub-bands, as expressed in Eq. (11).

11 $$\begin{aligned} R^t = -\sum _{j=1}^{M^t} r_j^t \log r_j^t \end{aligned}$$

where, $R^t$ denotes the sub band, $r_j^t$ error function, and $M^t$ is the boosting outputs. Background noise is removed and feature contrast enhanced by applying shrinkage to the wavelet coefficients, as expressed in Eq. (12).

12 $$\begin{aligned} D = s(r, g, b) \end{aligned}$$

where, $D$ define the colour feature and $s(r,g,b)$ functions as a map between the red(r), green (g) and blue (b) colour components to the colour feature $D$. These features provide a robust and discriminative representation of herb leaf images and are subsequently fed into the CVSTGCN network for accurate and reliable herb species classification.

### Classification using complex value spatio-temporal graph convolutional neural network (CVSTGCN)

**Fig. 3:**
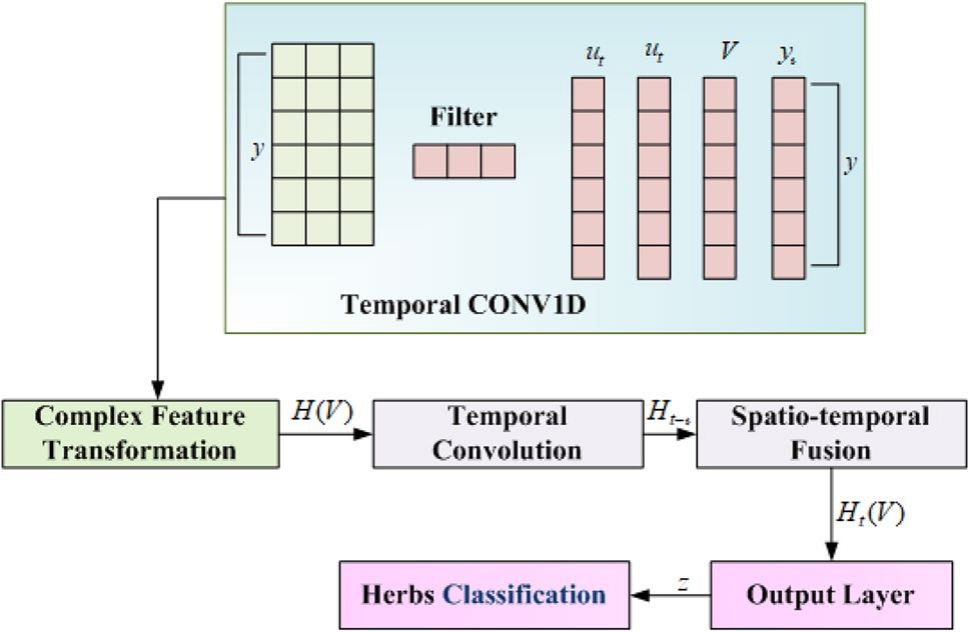
Architecture of CVSTGCN.

This section discusses the use of the CVSTGCN network^32^ for classifying herb images from both the FLAVIA and Medical Leaves datasets. The FLAVIA dataset includes species such as Tongkat Ali, Kacip Fatimah, Halia Rhizome, Pegaga, Patawali Stem, Kaduk Leaves, Ulam Raja Leaves, Akar Ginseng Jawa, Misai Kucing, Buah Kedondong, Dukung Anak, Sambung Nyawa Leaves, Citrosa, Ceri Barbados, Roselle, Pudina, Mulberi, Persicaria Perfoliata, Pokok Limau Kasturi, and Sedap Malam, while the Medical Leaves dataset consists of the following: Nyctanthes Arbor-tristis, Ocimum Tenuiflorum, Piper Betle, Plectranthus Amboinicus, Pongamia Pinnata, Psidium Guajava, Punica Granatum, Santalum Album, Syzygium Cumini, Syzygium Jambos, Tabernaemontana Divaricata, Citrus Limon, Ficus Auriculata, Ficus Religiosa, Hibiscus Rosasinensis, Jasminum, Mangifera Indica, Mentha, Moringa Oleifera, Muntingia Calabura, Murraya Koenigii, Nerium Oleander, Jasminum. The Architecture of CVSTGCN is shown in Fig. 3.

In the proposed CVSTGCN framework, the phase component of complex-valued features encodes the spatial orientation of patterns within the leaf image, while the magnitude captures the intensity of these patterns. Specifically, leaf veins form directional structures that define the leaf’s morphology. The phase represents how these vein structures are aligned and oriented relative to each other, for instance, whether veins radiate from the petiole at a certain angle or form parallel networks. By preserving this orientation information, the phase enables the network to distinguish leaves with similar textures or shapes but different vein arrangements, effectively capturing the directionality and structural patterns of leaf veins, which are critical morphological traits for herb species identification. The CVSTGCN network is selected for its ability to simultaneously capture magnitude and phase information in complex-valued feature representations, providing richer and more discriminative features than traditional real-valued CNNs. Temporal convolution and spatio-temporal fusion modules enable the network to model sequential dependencies and integrate spatial-temporal patterns, which is effective for distinguishing visually similar herb species.

The classification begins with a complex feature transformation of the input images from datasets, encoding magnitude and phase information of visual features such as color, shape, and texture, as expressed in Eq. (13).

13 $$\begin{aligned} G(R) = \sum _{j=0}^{J-1} g_j R^j \end{aligned}$$

Here, $G(R)$ represents the linear matrix operator, $R$ denotes the matrix operator, $J$ represents the length of the graph, and $g_j$ represents the real scalar value. The transformed features are structured into a spatio-temporal graph, where nodes represent image regions and edges represent spatial and temporal relationships, capturing local and global dependencies is given in Eq. (14).

14 $$\begin{aligned} v_s&= \sum _{q=0}^{s} G_{s - \tau }(R)\, w_\tau ,\quad G_s(R) =\sum _{j=0}^{J-1} g_{j,s} R^j \end{aligned}$$

Here, $v_s$ represents the element multiplication of the graph filter in times, R denotes the matrix operator, $G_{s-\tau }(R)$ represents the linear matrix operator, $w_\tau$ represents the graph point, $J$ represents the length of the graph and $g_{j,s}$ represents the real scalar value in times. The transformed features are structured into a spatio-temporal graph, where nodes represent image regions and edges represent spatial and temporal relationships, capturing local and global dependencies is given in Eq. (15).

15 $$\begin{aligned} G(R \otimes y)=\sum _{j=0}^{J-1} G_j(y) R^j,\quad G_j(y)=\sum _{s=0}^{J_s-1} g_{j,s} y^{-s} \end{aligned}$$

Here, $G(R)$ represents the linear matrix operator, $R$ denotes the matrix operator, $J$ represents the length of graph, $g_j$ represents the real scalar value, $y$ represents the transform and $\otimes$ denotes the tensor product. The second and third CVGC layers capture regional and global interactions to learn hierarchical patterns for discriminative feature extraction. The FLAVIA dataset images are classified using Eq. (16).

16 $$\begin{aligned} V(y) = G(R \otimes y)\, W(y) \end{aligned}$$

Here, $V$ represent the multiple channel output, $y$ represent the transform, $G(R)$represent the linear matrix operator, $W$ denote the multiple channel, and $\otimes$ denote the tensor product. The Medical Leaves dataset images are classified using Eq. (17).

17 $$\begin{aligned} \mathcal {H}(\Lambda , y)&= \sum _{s=0}^{J_s - 1} \sum _{j=0}^{J - 1} g_{j,s}\, \Lambda ^j\, y^{-s},\quad \bar{V}(y) = \mathcal {H}(\Lambda , y)\, \bar{W}(y) \end{aligned}$$

Here, $\mathcal {H}(\Lambda , y)$ represents the diagonal matrix, $g_{j-s}$ represents the real scalar value, $y$ represents the transform, $W$ denote the multiple channel and $V$ represents the multiple channeloutput is represented. Finally, the output layer applies softmax activation to generate probability scores for each class, with the highest probability selected as the predicted label is given in Eq. (18).

18 $$\begin{aligned} \bar{v} = \sigma [v] = \sigma \left[ \sum _{j=0}^{J-1} g_j R^j w\right] \end{aligned}$$

Here, $\bar{v}$ represents the element multiplication of the graph filter, $\sigma (\cdot )$ represents the input complex values, $g_j$ represents the real scalar value and $R$ denotes the matrix operator, a specific instance of multiple features. Finally, the class probabilities and predicted label are defined as Eqs. (19) and(20).

19 $$\begin{aligned} P(c \mid x)= & \frac{\exp (\bar{v}_{c})}{\displaystyle \sum _{k=1}^{C} \exp (\bar{v}_{k})} \end{aligned}$$

20 $$\begin{aligned} \hat{y}= & \arg \max _{c} \, P(c \mid x) \end{aligned}$$

Where, $P(c \mid x)$ is the probability of class *c* given the input *x*, $\bar{v}_{c}$ is the CVSTGCN output for class *c*, *C* denotes the total number of classes, and $\hat{y}$ represents the predicted label. Finally, the CVSTGCN successfully classifies the herb species from both the FLAVIA and Medical Leaves datasets. In this work, HMRFO is utilized to optimize the optimum factors $v_{s}$ and $\bar{v}$. Here, HMRFO is employed to tune both the weight and bias factors of the model.

### Optimization using hierarchical manta ray foraging optimization (HMRFO)

This section discusses the optimization of proposed CVSTGCN using HMRFO^33^ to improve weight parameters $v_s$ and $\bar{v}$. The HMRFO algorithm was chosen for its superior ability to balance exploration and exploitation during model parameter tuning. Inspired by the intelligent foraging behavior of manta rays, it introduces a hierarchical structure that enhances the diversity of solutions and accelerates convergence toward the global optimum. Unlike traditional optimization methods, it dynamically adjusts search strategies across multiple layers to avoid local minima and improve robustness. This makes it particularly suitable for fine-tuning complex deep learning models like CVSTGCN, where parameter sensitivity directly impacts classification accuracy. Its adaptive nature and strong global search capability ensure improved optimization of network weights and learning rates, resulting in better generalization and performance. Step-by-step procedure of HMRFO optimization discussed as follows.

*Step 1: Initialization*

Randomness generates the initial populace of the HMRFO. Initialization is derived using Eq. (21) .

21 $$\begin{aligned} \textbf{C} = \begin{bmatrix} C_1^1 & C_1^2 & \cdots & C_1^E \\ C_2^1 & C_2^2 & \cdots & C_2^E \\ \vdots & \vdots & \ddots & \vdots \\ C_n^1 & C_n^2 & \cdots & C_n^E \end{bmatrix} \end{aligned}$$

Here, $C$ is denotes the matrix of prime populations and $E$ is denotes the $i ^ {th}$ individual’s objective function value.

*Step 2: Random generation*

After the initial setup, input parameters were randomly generated within predefined ranges to ensure diverse exploration of the solution space. Each generated set represents potential hyperparameters, which are evaluated based on their fitness values. This process helps identify the most suitable candidates for achieving optimal model performance.

*Step 3: Fitness function*

A random solution is produced from initialized values. It is computed through parameter optimization. Equation (22) is then used to derive the formula.

22 $$\begin{aligned} \text {FitnessFunction} = \operatorname {optimizing}(v_s, \bar{v}) \end{aligned}$$

The variables $v_s$ is employed for maximizing the accuracy and $\bar{v}$ is employed for increasing the ROC.

*Step 4: Cyclone foraging for optimizing* $v_s$

HMFO, cyclone foraging has various benefits through the utilization of hierarchical structures that are similar to the foraging behavior of manta rays, HMRF maximizes information collection within a cyclone, consequently improving efficiency. Adaptive adjustments and better resource utilization are possible with this approach, which makes it especially useful in environments that are changing quickly. Thus, it is given in Eq. (23)

23 $$\begin{aligned} B_j = \sqrt{(y_j^1 - y_{\text {best}}^2)^2 + (\theta - y_{\text {best}}^2)^2 + (y_j^b - y_{\text {best}}^b)^2 \cdot v_s} \end{aligned}$$

Here, $B_j$ indicates the gap between the greatest person and the $i^{th}$ individual; the partial derivatives with respect to $\theta$. Thus, it is given in Eq. (24)

24 $$\begin{aligned} \exists _{j=1}^{M} \; P_j = \delta \cdot E_j + (1 - \delta ) \cdot B_j \end{aligned}$$

where, $P_j$ indicates the ith individual’s score; $\delta$ represents the functional weights; and $\delta$ produced at random using a Gaussian distribution; $E_j$ denotes the fitness value; $B_j$ indicates the distance between the $i ^ {th}$ individual.

*Step 5: Chain foraging for optimizing* $\bar{v}$

Chain foraging optimizes the search process by striking a balance between local exploitation and global exploration. This hierarchical structure improves scalability by efficiently managing complex tasks by decomposing them into more manageable subtasks. HMRFO also has the advantage of being decentralized, which improves resilience and environment adaptability. This qualifies it for a variety of real-world uses requiring complex task management and resource allocation. Thus, it is given in Eq. (25)

25 $$\begin{aligned} y_j^b(v + 1) = y_j^b(\upsilon ) + P \cdot \left( s_2 \cdot y_{\text {RS1}}^b - s_2 \cdot y_j^b(\bar{v}) \right) \end{aligned}$$

Where, $y_j^b$ denotes updating the population; $\bar{v}$ denotes the attention scores assigned to neighbourhoods of different hops, $y_{RS1}^b$ denotes choosing at random a person from the top $RS1$ of the people sorted using the functional weight and fitness-distance balance selection method; $(v)$ represents the most iterations possible, $s$ represents random vector; Thus, it is given in Eq. (26)

26 $$\begin{aligned} g_j^b(e + 1) = g_j^b(w) + y \cdot \left( e_2 \cdot x_{\text {RS2}}^b - e_2 \cdot g_j^b(w) \right) \end{aligned}$$

Here, $g_j^b$ denotes updating the population; $x_{RS2}^b$ indicates the process of choosing at random a person from the top $RS1$ of the individuals sorted using functional weight and the fitness-distance balance selection technique; $(w)$ represents the most iterations possible; $e$ represents the random vector.

*Step 6: Update best solution*

After each iteration, the global best solution is updated to guide the next generation is given in Eq. (27).

27 $$\begin{aligned} Y_{\text {Best}} = \arg \max _{C_i}(C_i) \end{aligned}$$

Where, $Y_{\text {Best}}$ represents the solution with the highest fitness score among all individuals. This ensures that the algorithm converges toward the optimal configuration of weight and attention parameters.

*Step 7: Termination*

Until the position data is acquired, theweight factor $v_s$ and $\bar{v}$ from the neural network optimized with HMRFO will continue. Then AHC-CVSTGCN is used for herbs classification for optimizing the accuracy and ROC.

### Time complexity of CVSTGCN-HMRFO

To evaluate the computational efficiency of the proposed framework, this section analyzes the time complexity of both the CVSTGCN network and the HMRFO optimization algorithm. Understanding the time complexity helps in estimating the feasibility and scalability of the method for large-scale herbal classification tasks. The time complexity of the CVSTGCN network is given in Eq. (28).

28 $$\begin{aligned} O_{\text {CVSTGCN}} = O\!\left( L \cdot (V + E) \cdot C_{\text {in}} \cdot C_{\text {out}} \;+\; V \cdot C_{\text {out}} \cdot K \right) \end{aligned}$$

Here, *V* is denoted as the number of nodes, *E* is indicated as the number of edges, $C_{\text {in}}$ and $C_{\text {out}}$ represent the input and output channels per layer, *L* is denoted as the number of graph convolution layers, and *K* is the number of output classes. The time complexity of the HMRFO algorithm is expressed in Eq. (29).

29 $$\begin{aligned} O_{\text {HMRFO}} = O\!\left( T_{\max } \cdot n \cdot d \right) \end{aligned}$$

Where, $T_{\max }$ is denoted as the maximum number of iterations, *n* is indicated as the number of search agents, and *d* is indicated as the number of hyperparameters to optimize. The overall time complexity of the proposed framework is calculated using Eq. (30)

30 $$\begin{aligned} O_{\text {Total}} = O_{\text {CVSTGCN}} + O_{\text {HMRFO}} \end{aligned}$$

The total complexity accounts for both graph-based spatio-temporal feature learning and the optimization process, scaling linearly with the amount of nodes, edges, temporal steps, channels, and hyperparameters.

## Results and discussion

This section discusses the results for the experimental conduct of the proposed AHC-CVSTGCN technique. The system for conducting the experiment is equipped with a Windows operating system installed, Intel Core i3-3220 CPU operates on 64 bit with a 3.30 GHz clock speed and supported by 4.00 GB of installed RAM with 3.89 GB usable. The 70:30 split ratio is used for training and testing purposes respectively. The proposed AHC-CVSTGCN performance is evaluated and compared with existing techniques, including such as textural analysis for medicinal plant identification using log gabor filters (TA-MPI-LGF-KNN)^21^, an effective and automated method for classifying herbs based on shape and texture features using deep learning (AHC-ST-DLNN)^22^, and a random forest classifier employed for modeling and classification of herbal plants considering various features utilizing machine learning (MHPDF-RF)^23^. Table 2 summarizes the hyperparameter settings used for configuring the proposed AHC-CVSTGCN model. A learning rate of 0.001 guarantees stable convergence during training, and the ReLU activation function is used to introduce non-linearity for the CVSTGCN network. For computational efficiency, a batch size of 64 is employed, and overfitting is avoided by applying a dropout rate of 0.3. To get the best results, the model is trained over 200 epochs. In order to properly optimize the network, the parameters for the HMRFO algorithm are 100 iterations, a population size of 30, and 10 search agents. The search domain is set within the range [0,1].

**Table 2:** Hyperparameter settings of AHC-CVSTGCN.

Parameter	Values
CVSTGCN	
Activation function	ReLU
Learning rate	0.001
Batch size	64
Dropout rate	0.3
Epochs	200
HMRFO	
Number of iterations	100
Population size	30
Number of search agents	10
Search domain	[0, 1]

### Performance measures

Performance metrics like accuracy, precision, recall, specificity, true positive rate, FPR, FNR, F-measure, and ROC are used to assess performance and are discussed.

#### Accuracy

To get the accuracy value given in Eq. (31), the ratio of the count of samples properly categorized per scheme to the entire count of samples is used.

31 $$\begin{aligned} \text {Accuracy} = \frac{TP + TN}{TP + TN + FP + FN} \end{aligned}$$

Here, $TP$ implies true positive, $TN$ implies true negative, $FP$ signifies false positive, and $FN$ represents false negative.

#### Precision

Precision evaluates how well the model distinguishes relevant positive cases from irrelevant ones. It is defined in Eq. (32) as the ratio of true positives to all predicted positives, showing the effectiveness of the model in minimizing false positives.

32 $$\begin{aligned} \text {Precision} = \frac{TP}{TP + FP} \end{aligned}$$

#### Recall

Recall is calculated by dividing the total number of true positive and false negative predictions by the number of true positives. Equation (33) shows the model’s ability to collect all relevant instances.

33 $$\begin{aligned} \text {Recall} = \frac{TP}{TP + FN} \end{aligned}$$

#### F-measure

It uses precision and recall to assess the model’s accuracy, which is important when dealing with unbalanced data and as per Eq. (34).

34 $$\begin{aligned} \text {F1-Score} = \frac{2 \times (\text {Precision} \times \text {Recall})}{\text {Precision} + \text {Recall}} \end{aligned}$$

#### Specificity

The specificity of a test refers to how effectively it can distinguish healthy occurrences. To estimate it, the percentage of genuine negatives in healthy occurrences must first be calculated. Specificity represented in Eq. (35)

35 $$\begin{aligned} \text {Specificity} = \frac{TN}{TN + FP} \end{aligned}$$

#### False positive rate

The false positive rate is the rate at which items from the negative class are mistakenly classified as belonging to the positive class by the classifier. False positive rate represented in Eq. (36)

36 $$\begin{aligned} \text {False Positive Rate (FPR)} = \frac{\text {FP}}{\text {FP} + \text {TN}} \end{aligned}$$

#### False negative rate

The FNR refers to how often the classifier incorrectly allocates objects from the positive class to the negative class, as represented in Eq. (37).

37 $$\begin{aligned} \text {False Negative Rate (FNR)} = \frac{\text {FN}}{\text {FN} + \text {TP}} \end{aligned}$$

#### True positive rate

The proportion of real positive instances that the classification method or test accurately classified as positive is known as the TPR as represented in Eq. (38).

38 $$\begin{aligned} \text {True Positive Rate (TPR)} = \frac{\text {TP}}{\text {TP} + \text {FN}} \end{aligned}$$

#### Receiver operating characteristic (ROC)

In terms of graphics, ROC may be defined as the ratio of one variable’s changes to those of another; a line’s slope indicates the rate of change and represented in Eq. (39)

39 $$\begin{aligned} \text {ROC} = 0.5* \left( \frac{\text {TP}}{\text {TP} + \text {FN}} + \frac{\text {TN}}{\text {TN} + \text {FP}} \right) \end{aligned}$$

### Performance analysis

This section discusses the simulation results of the proposed AHC-CVSTGCN technique and is depicted in Fig. 5, 6, 7, 8, 9, 10, 11, 12 and 13. Accuracy, precision, recall, F-measure, specificity, FPR, FNR, TPR, and ROC are performance metrics used to evaluate performance.

#### Performance analysis for FLAVIA dataset

**Fig. 4:**
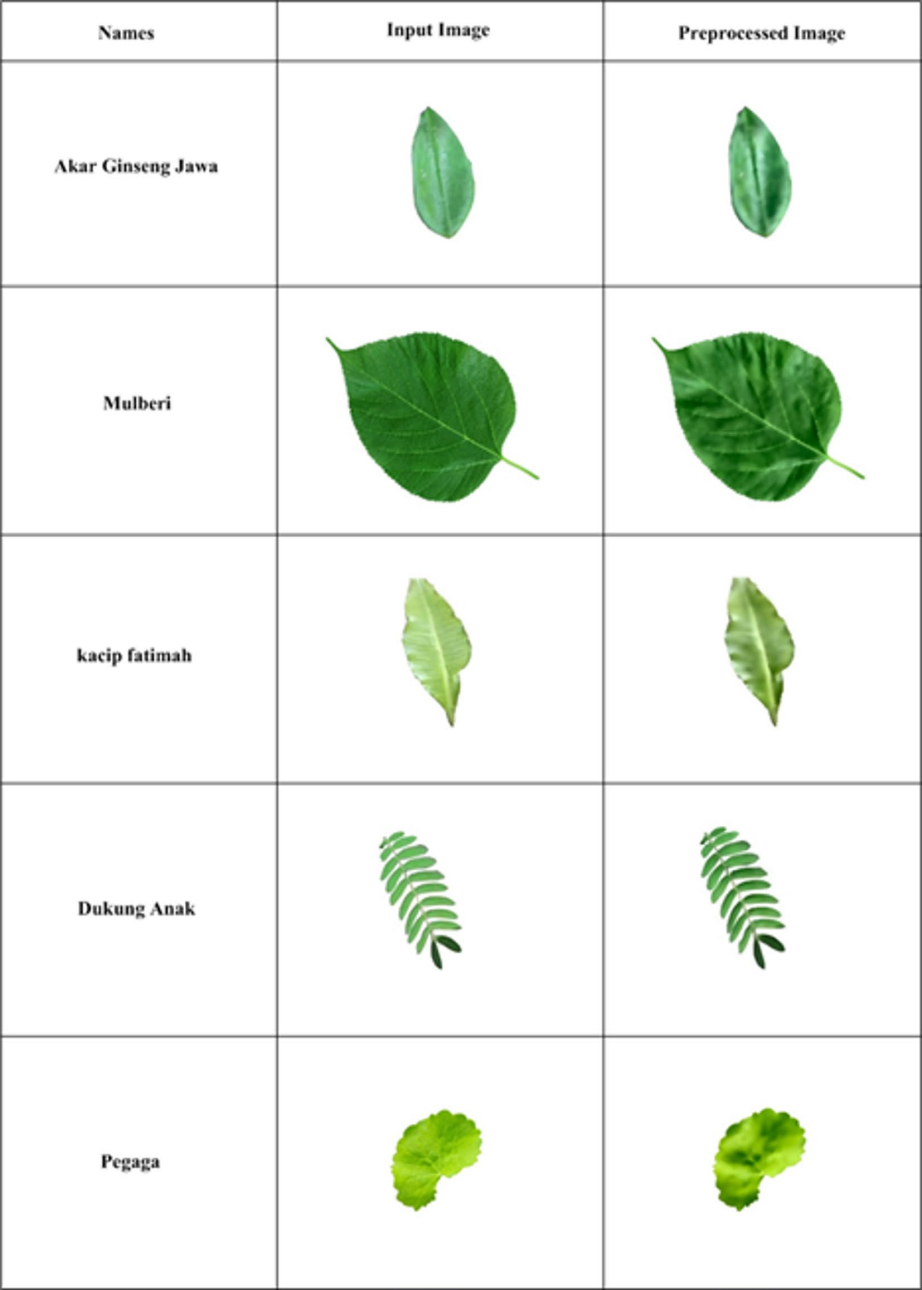
Input and preprocessed images.

Figure 4 compares raw leaf images and preprocessed images and indicate that the preprocessing steps like resizing, normalization, noise reduction and color correction allow preserving structural and textural information to be analyzed. The five example classes including Akar Ginseng Jawa, Mulberi, Kacip Fatimah, Dukung Anak and Pegaga demonstrate the variation of shape, size and texture and how the preprocessing standardizes images to allow correct classification among the 20 classes dataset.

**Fig.5:**
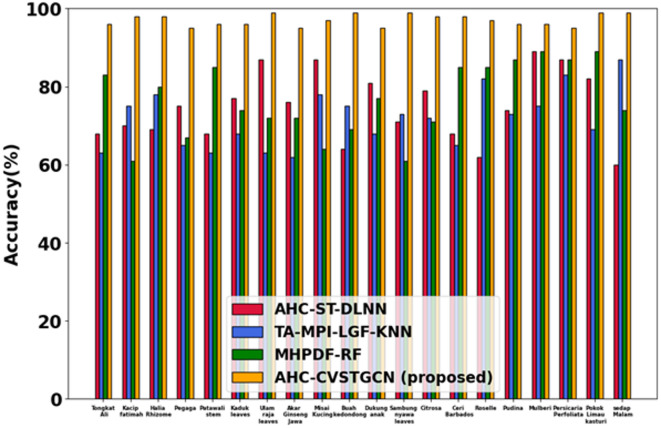
Evaluation of accuracy.

Figure 5 displays the analysis of accuracy performance. displays the accuracy analysis. Tongkat Ali, Halia Bara, Patawali Stem, Kaduk Leaves, 98.14%, 96.47%, 94.56%, Cerbera Odollam, Cerbera Manghas, 98.12%, Simpoh Air, 98.41%, 92.71%, 96.21%, Pegaga, 99.8%, Halia Rhizome, 98.57%, Kacip Fatimah, 98.13%, Mas Cotek, 99.7%, Pokok Limau Kasturi, 99.1%, Tongkat Ali Root, 98.45%, 98.29%, Lemon Grass, and 98.67% are all achieved by the AHC-CVSTGCN. Because CVSTGCN successfully captures both temporal and spatial dependencies, the accuracy is high.

**Fig. 6:**
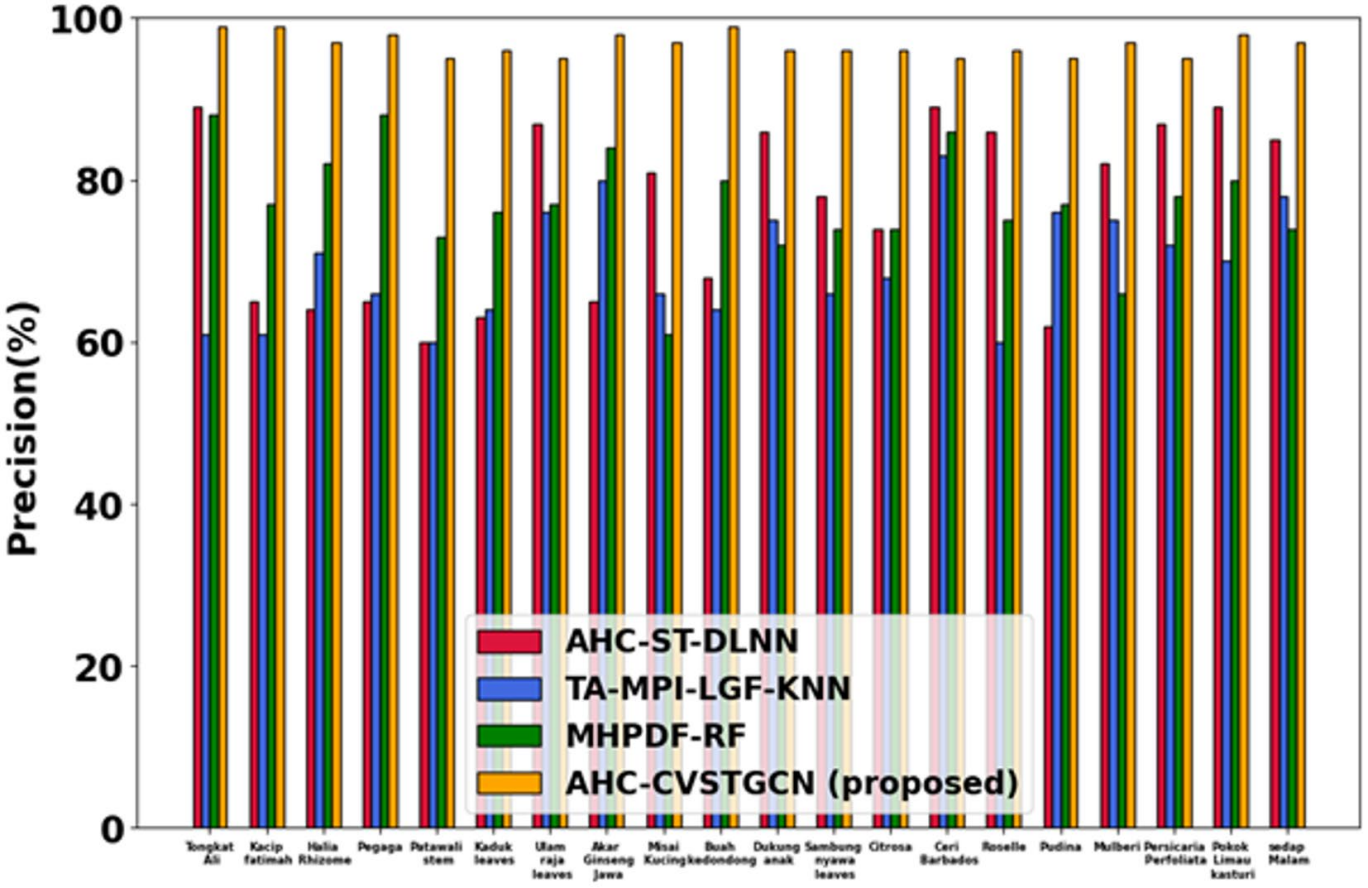
Evaluation of precision.

Figure 6 displays the evaluation of precision performance for the proposed AHC-CVSTGCN model. Tongkat Ali, Kacip Fatimah, Halia Rhizome, Pegaga, Patawali Stem, Kaduk Leaves, Ulam Raja Leaves, Akar Ginseng Jawa, Misai Kucing, Buah Kedondong, 98.65%, Dukung Anak, 99.10%, Sambung Nyawa Leaves, 97.05%, Citrosa, Ceri Barbados, 90.60%, Roselle, 95.05%, Mulberi, 98.80%, Persicaria Perfoliata, Pokok Limau Kasturi, and Sedap Malam are all achieved by the model. By lowering false positives, HMRFO optimization increases the reliability of positive predictions.

**Fig. 7:**
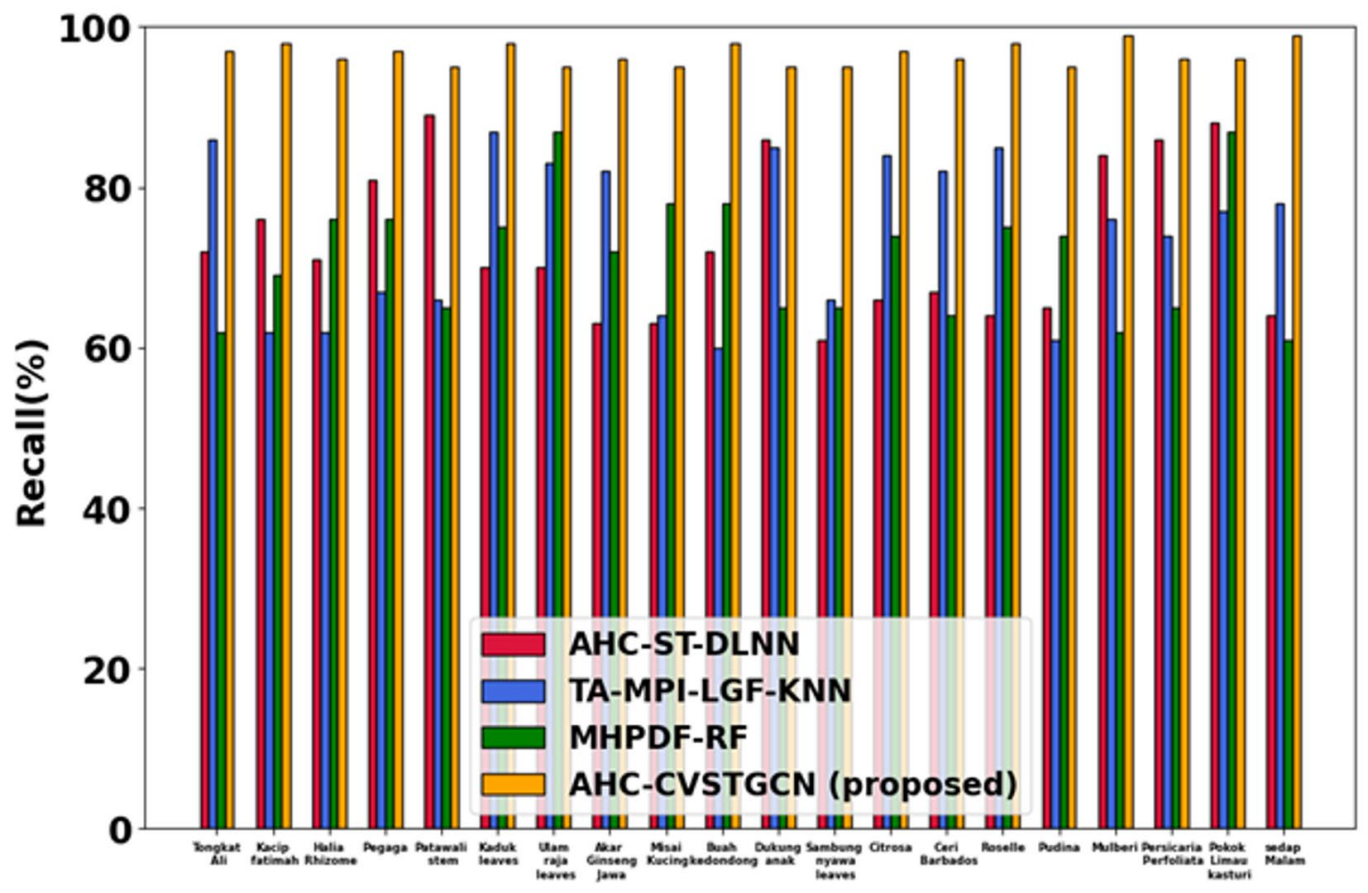
Evaluation of recall.

Figure 7 depicts the evaluation of recall. The AHC-CVSTGCN achieves 97.16% for Tongkat Ali, 96.09% for Kacip Fatimah, 98.67% for Halia Rhizome, 98.80% for Pegaga, 98.05% for Patawali Stem, 99.12% for Kaduk Leaves, 99.14% for Ulam Raja Leaves, 99.09% for Akar Ginseng Jawa, 98.07% for Misai Kucing, 98.22% for Buah Kedondong, 97.65% for Dukung Anak, 98.60% for Sambung Nyawa Leaves, 99.05% for Citrosa, 98.37% for Ceri Barbados, 99.10% for Roselle, 99.05% for Pudina, 98.42% for Mulberi, 98.80% for Persicaria Perfoliata, 99.02% for Pokok Limau Kasturi, and 97.07% for Sedap Malam. MLPF preprocessing ensures critical features are preserved, reducing missed positives.

**Fig. 8:**
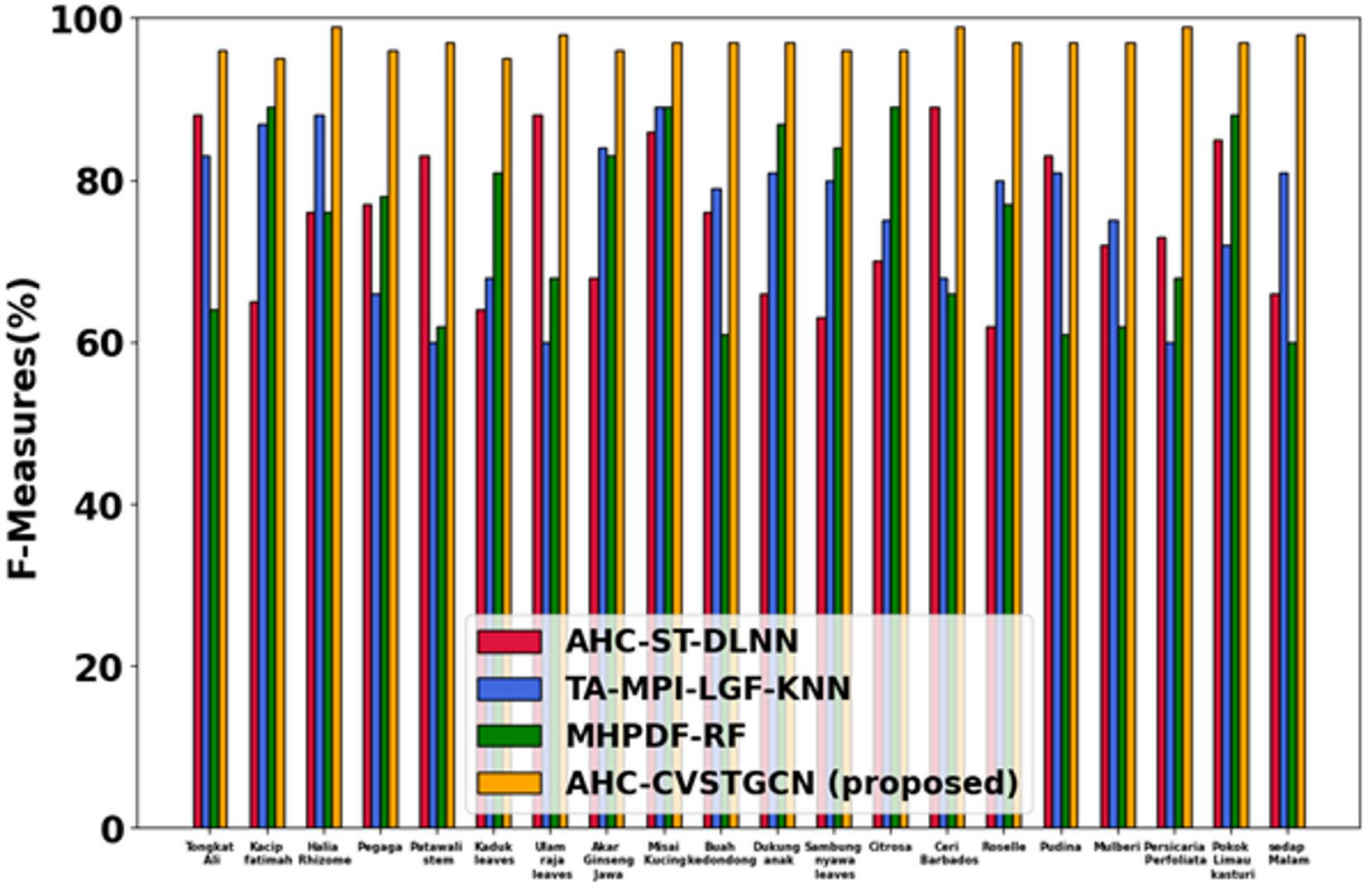
Evaluation of F1-measure.

Figure 8 displays the evaluation of the F1-measure. The model achieves 96.11% for Tongkat Ali, 98.42% for Kacip Fatimah, 94.67% for Halia Rhizome, 98.80% for Pegaga, 99.05% for Patawali Stem, 97.67% for Kaduk Leaves, 96.89% for Ulam Raja Leaves, 99.09% for Akar Ginseng Jawa, 98.07% for Misai Kucing, 98.52% for Buah Kedondong, 96.65% for Dukung Anak, 98.70% for Sambung Nyawa Leaves, 99.05% for Citrosa, 94.37% for Ceri Barbados, 98.60% for Roselle, 99.15% for Pudina, 98.67% for Mulberi, 99.00% for Persicaria Perfoliata, 99.09% for Pokok Limau Kasturi, and 96.07% for Sedap Malam. High F1 reflects the balance between recall and precision due to the combination of RQFWT and HMRFO optimization.

**Fig. 9:**
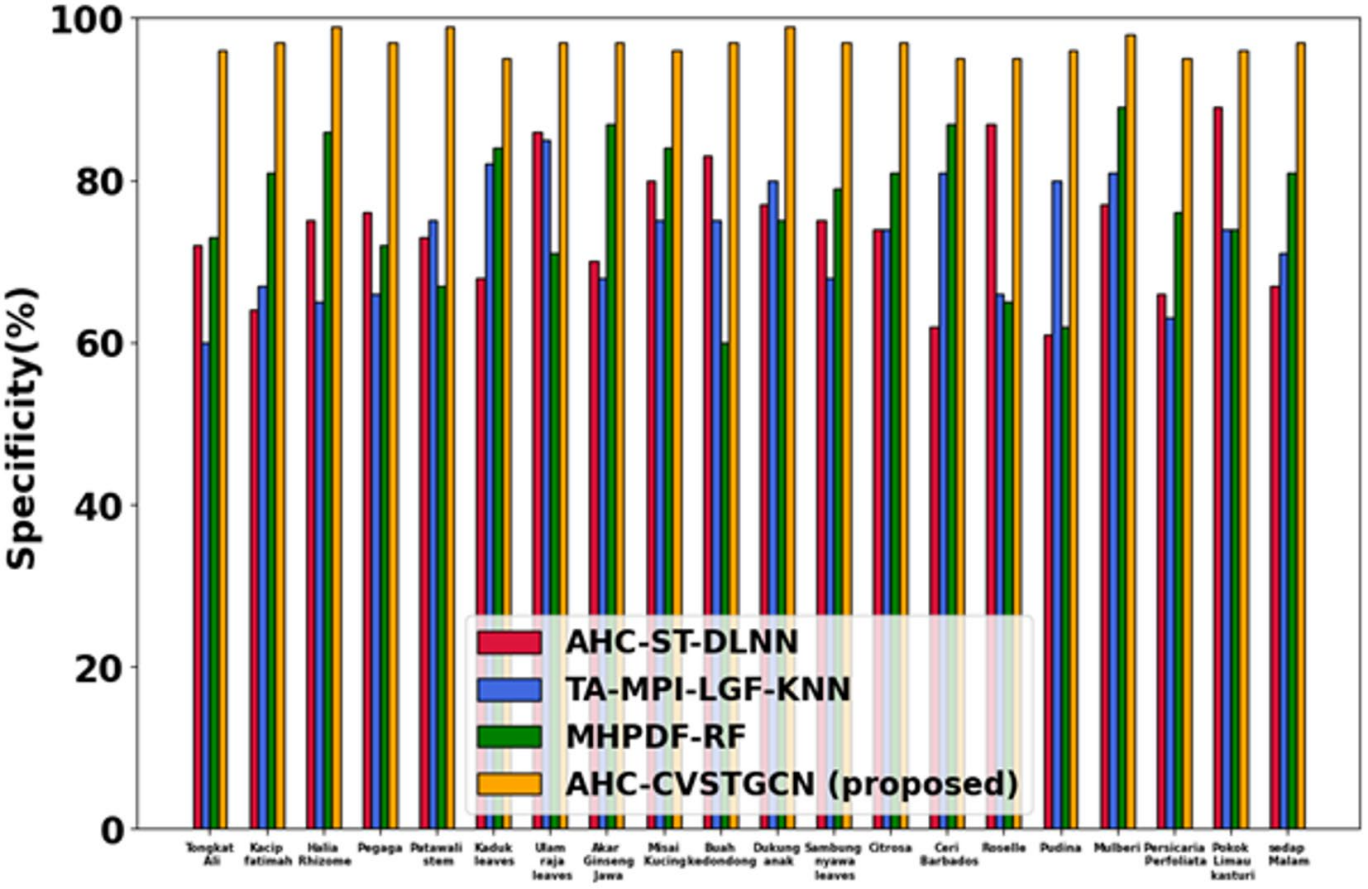
Evaluation of specificity.

Figure 9 depicts the evaluation of specificity. Tongkat Ali has a specificity of 96.01%, Kacip Fatimah has a 97.42%, Halia Rhizome has a 98.67%, Pegaga has a 95.80%, Patawali Stem has a 99.15%, Kaduk Leaves has a 99.21%, Ulam Raja Leaves has a 98.89%, Akar Ginseng Jawa has a 99.09%, Misai Kucing is 98.07%, Buah Kedondong has a 98.52%, Dukung Anak has a 95.55%, Sambung Nyawa leaves have a 98.70%, Citrosa has a 98.37%, Ceri Barbados has a 99.00%, Pudina has a 97.05%, Mulberi has a 98.67%, Persicaria has a 98.80%, Pokok Limau Kasturi has a 99.09%, and Sedap Malam is 98.55%. MLPF noise reduction aids in achieving high specificity.

**Fig. 10:**
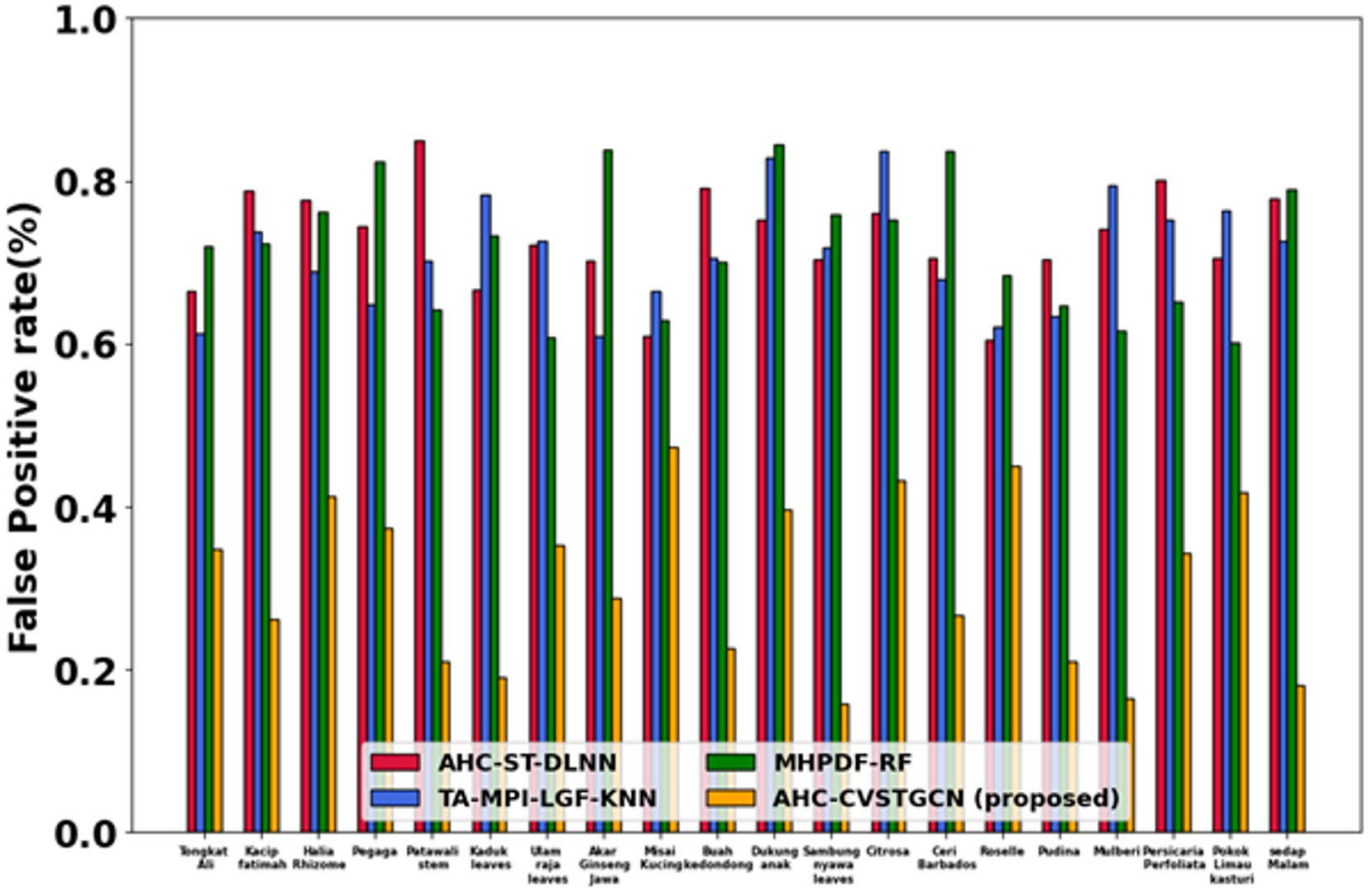
Evaluation of FPR.

Figure 10 depicts the performance analysis of the false-positive rate (FPR). False-positive rates are very low: 0.34% for Tongkat Ali, 0.26% for Kacip Fatimah, 0.41% for Halia Rhizome, 0.37% for Pegaga, 0.21% for Patawali Stem, 0.35% for Kaduk Leaves, 0.38% for Ulam Raja Leaves, 0.29% for Akar Ginseng Jawa, 0.47% for Misai Kucing, 0.23% for Buah Kedondong, 0.39% for Dukung Anak, 0.18% for Sambung Nyawa Leaves, 0.42% for Citrosa, 0.24% for Ceri Barbados, 0.42% for Roselle, 0.20% for Pudina, 0.18% for Mulberi, 0.30% for Persicaria Perfoliata, 0.41% for Pokok Limau Kasturi, and 0.18% for Sedap Malam. Low FPR results from HMRFO improving decision boundaries.

**Fig. 11:**
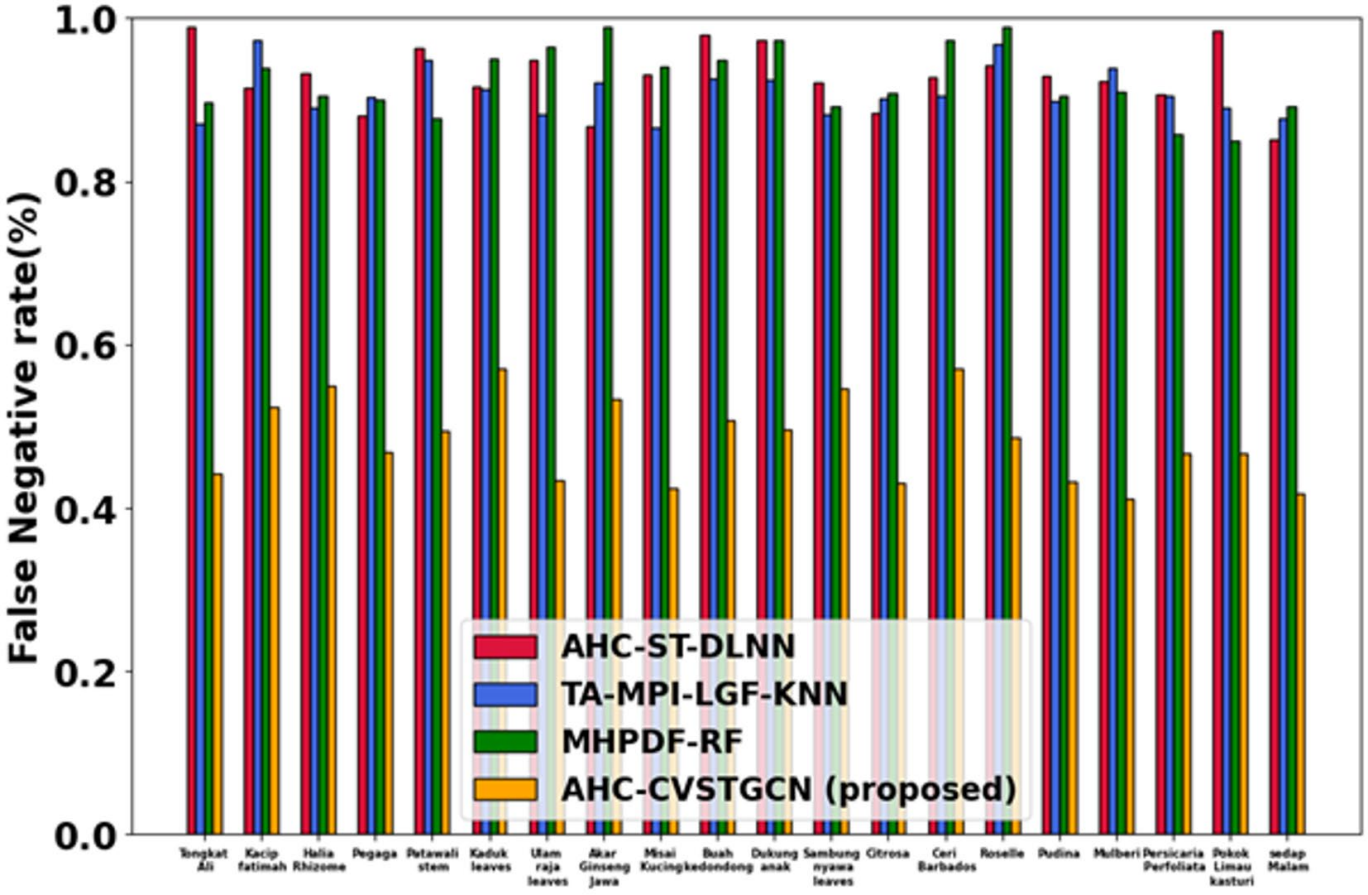
Evaluation of FNR.

Figure 11 depicts the evaluation of the false-negative rate (FNR). False-negative rates are minimal: 0.45% for Tongkat Ali, 0.53% for Kacip Fatimah, 0.52% for Halia Rhizome, 0.50% for Pegaga, 0.53% for Patawali Stem, 0.55% for Kaduk Leaves, 0.45% for Ulam Raja Leaves, 0.50% for Akar Ginseng Jawa, 0.45% for Misai Kucing, 0.50% for Buah Kedondong, 0.50% for Dukung Anak, 0.55% for Sambung Nyawa Leaves, 0.43% for Citrosa, 0.52% for Ceri Barbados, 0.45% for Roselle, 0.42% for Pudina, 0.52% for Mulberi, 0.42% for Persicaria Perfoliata, 0.42% for Pokok Limau Kasturi, and 0.46% for Sedap Malam. Low FNR ensures minimal missed classifications.

**Fig. 12:**
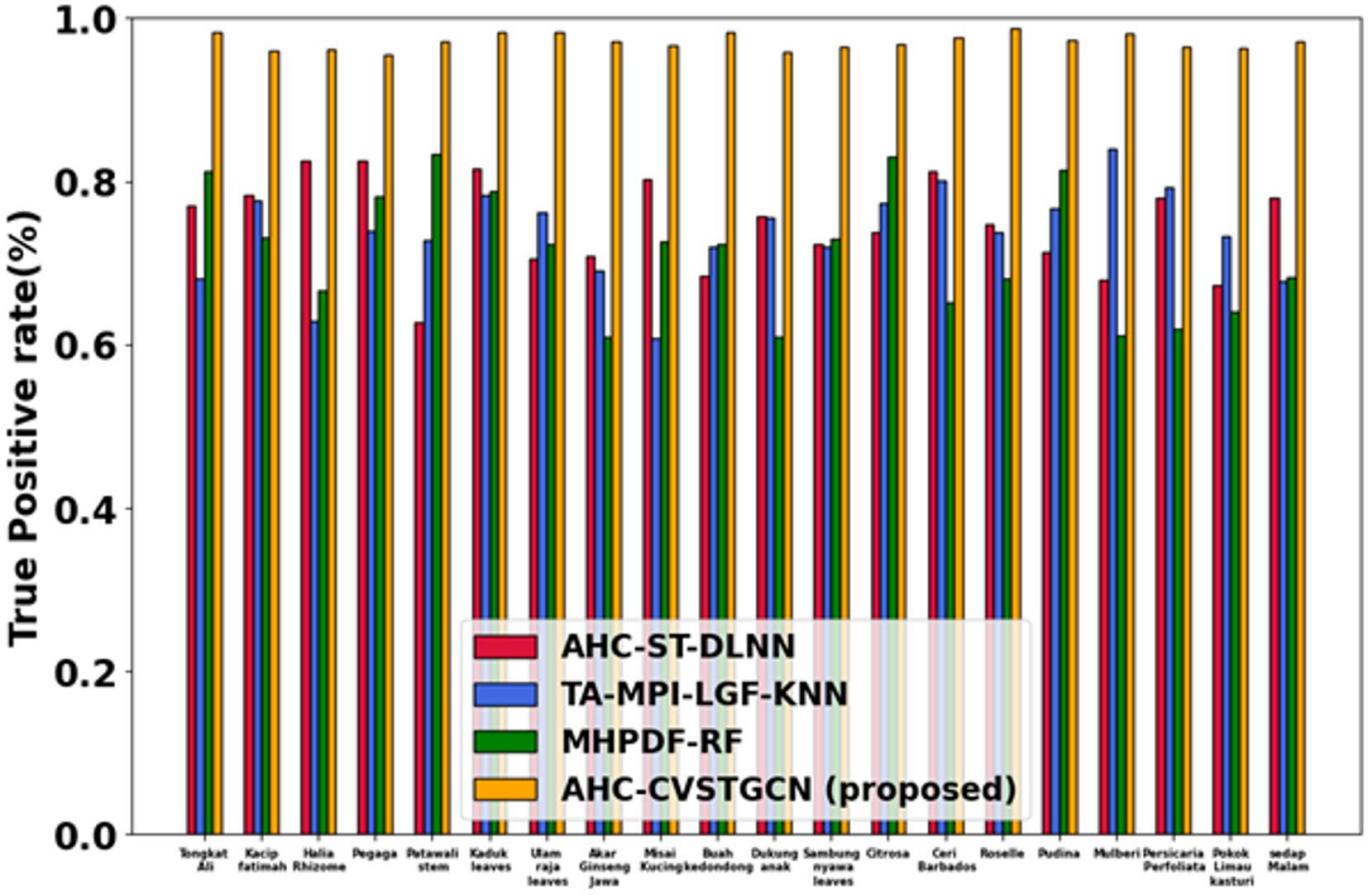
Evaluation of TPR.

Figure 12 depicts the performance analysis of the true positive rate (TPR). True positive rates are very high: 0.99 for Tongkat Ali, 0.99 for Kacip Fatimah, 0.95 for Halia Rhizome, 0.96 for Pegaga, 0.97 for Patawali Stem, 0.96 for Kaduk Leaves, 0.89 for Ulam Raja Leaves, 0.99 for Akar Ginseng Jawa, 0.96 for Misai Kucing, 0.97 for Buah Kedondong, 0.95 for Dukung Anak, 0.80 for Sambung Nyawa Leaves, 0.85 for Citrosa, 0.92 for Ceri Barbados, 0.93 for Roselle, 0.97 for Pudina, 0.98 for Mulberi, 0.90 for Persicaria Perfoliata, 0.94 for Pokok Limau Kasturi, and 0.97 for Sedap Malam. High TPR reflects the accurate detection ability of CVSTGCN with optimized features.

**Figure 13:**
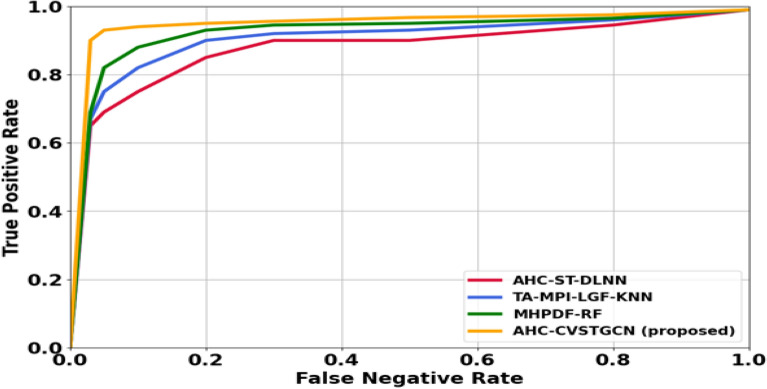
Evaluation of ROC.

Figure 13 illustrates the ROC-AUC comparison of the proposed and existing models. The proposed AHC-CVSTGCN achieves the highest AUC of 0.9637, indicating excellent class discrimination capability. In comparison, MHPDF-RF attains an AUC of 0.9266, while TA-MPI-LGF-KNN and AHC-ST-DLNN achieve 0.9084 and 0.8961, respectively. The ROC curve of AHC-CVSTGCN rises more steeply toward the top-left corner, showing a higher true positive rate at lower false positive rates, which confirms its superior classification reliability over the competing methods.

**Fig. 14:**
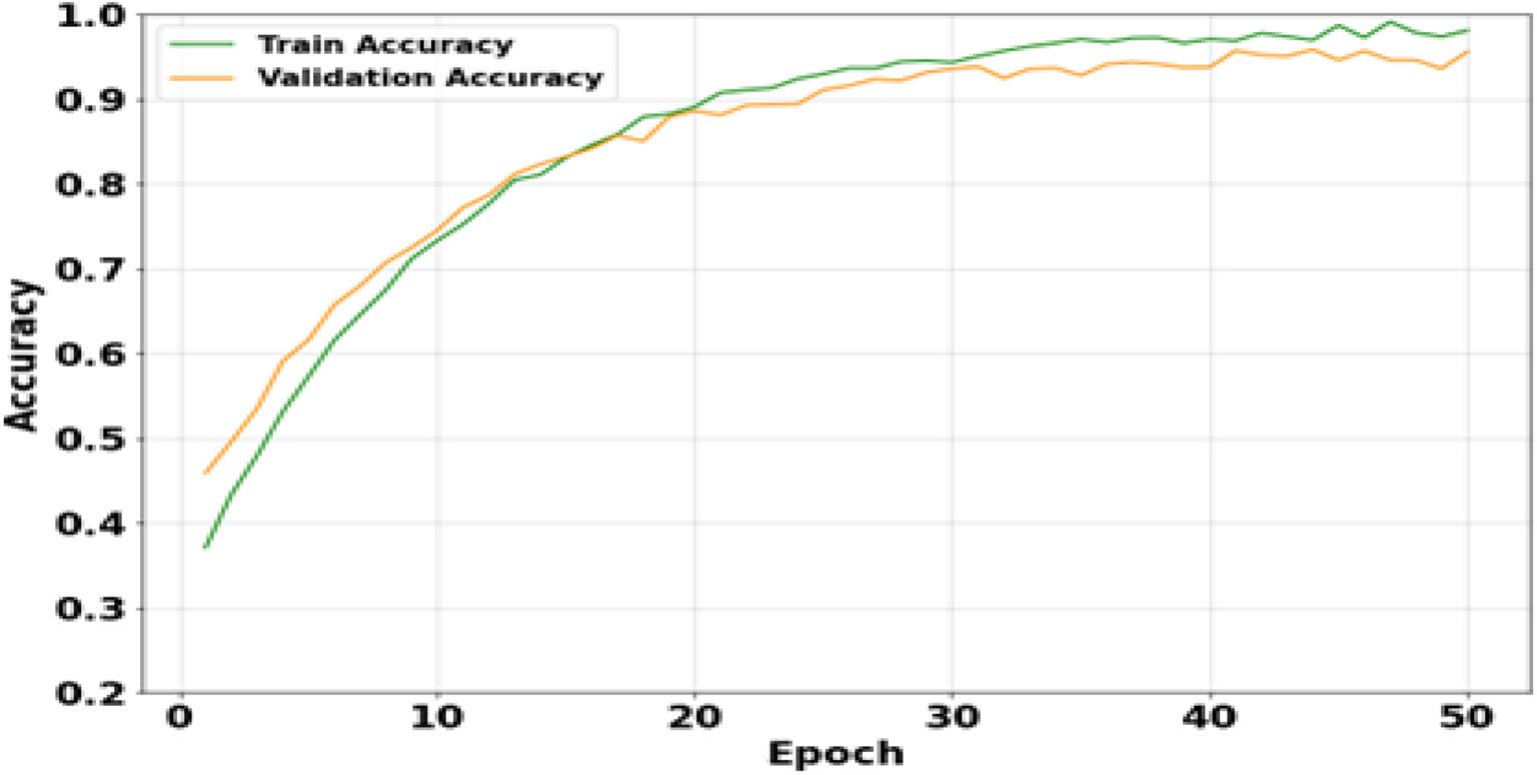
Analysis of accuracy.

Figure 14 shows the accuracy progression over 50 epochs. Training accuracy improves from about 37% to nearly 99%, while validation accuracy increases from 46% and stabilizes between 94-96% after Epoch 30. The small gap indicates mild overfitting, but the high validation accuracy confirms strong generalization. The consistently better results are due to robust feature extraction, CVSTGCN spatial-temporal learning, and optimized HMRFO parameters, allowing accurate classification across all species.

**Fig. 15:**
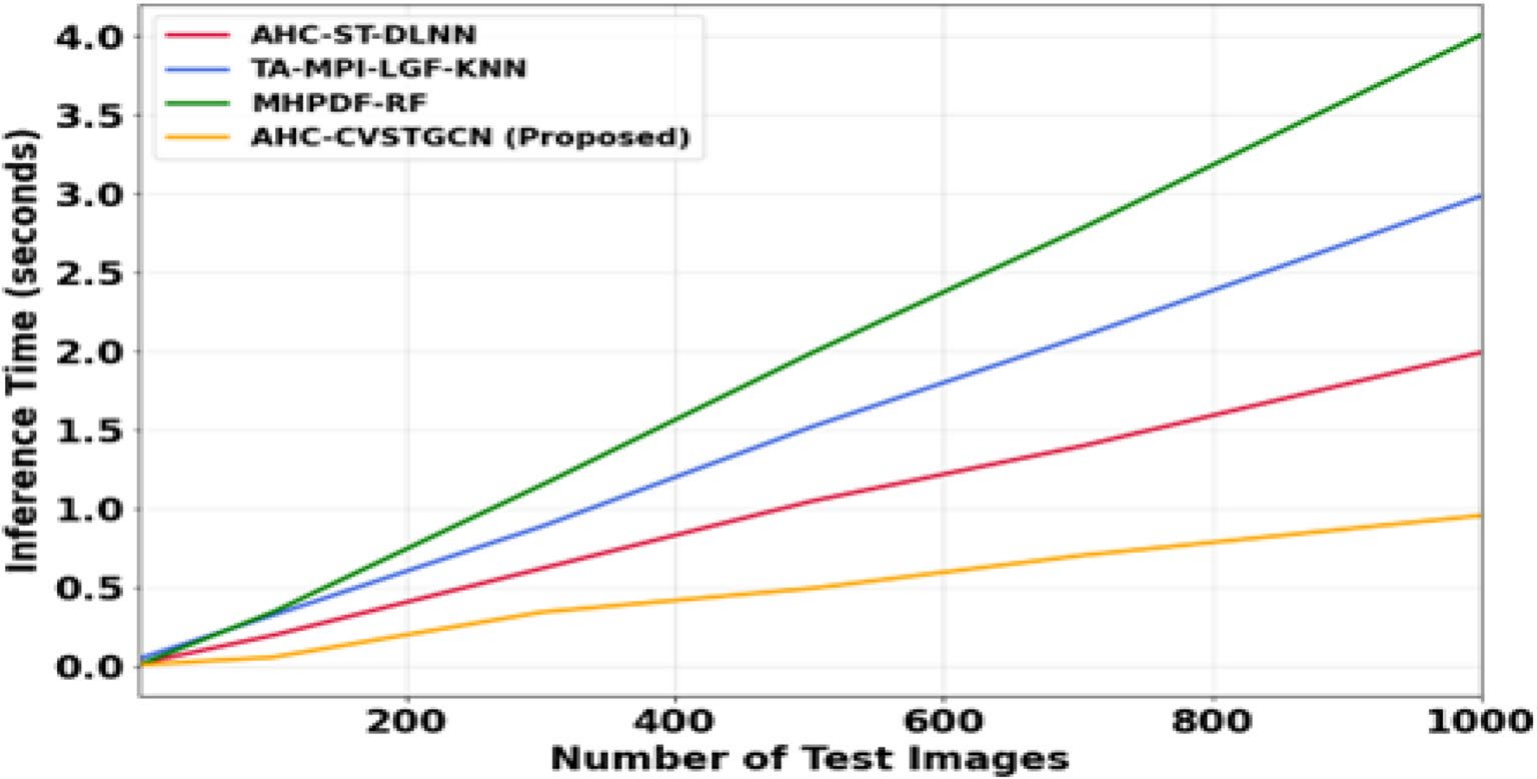
Analysis of computational scalability of FLAVIA dataset.

Figure 15 the computational scalability of different models on the FLAVIA dataset is presented. As the number of test images increases to 1000, the MHPDF–RF model requires approximately 4.0 s, TA–MPI–LGF–KNN requires 3.0 s, and AHC–ST–DLNN requires 2.0 s, whereas the proposed AHC–CVSTGCN achieves an inference time of less than 1.0 s. The significantly faster performance of the proposed model is attributed to its optimized CVSTGCN architecture and the integration of HMRFO, which effectively reduces redundant computations while maintaining high classification accuracy.

**Fig. 16:**
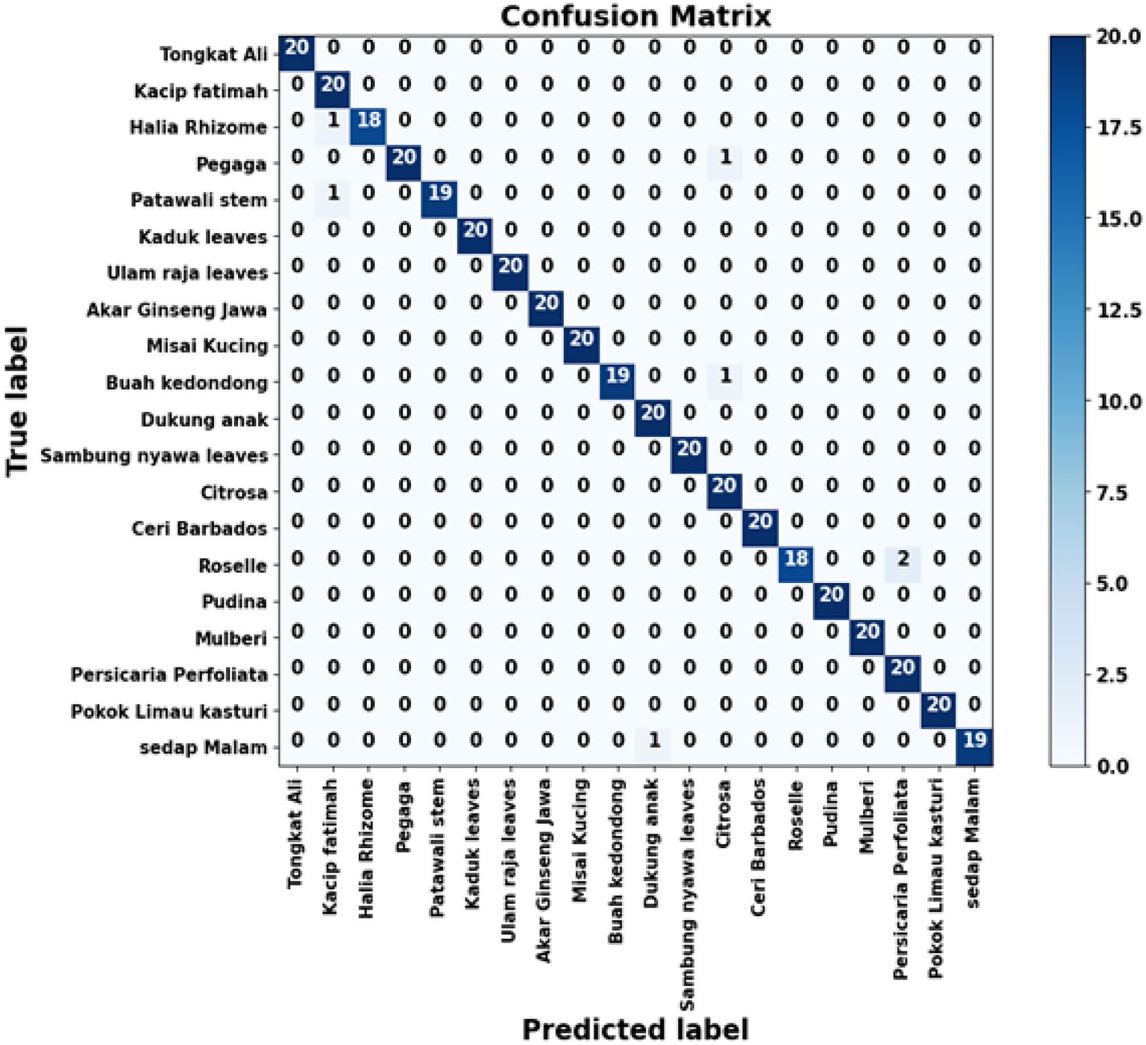
Confusion matrix of FLAVIA dataset.

Figure 16 shows a strong diagonal pattern in the confusion matrix, indicating that almost all herbal leaf samples are correctly classified. Most classes achieve 19-20 correct predictions, with only a few minor misclassifications between visually similar species. This confirms the high accuracy and robustness of the proposed method on the FLAVIA dataset.

**Fig. 17:**
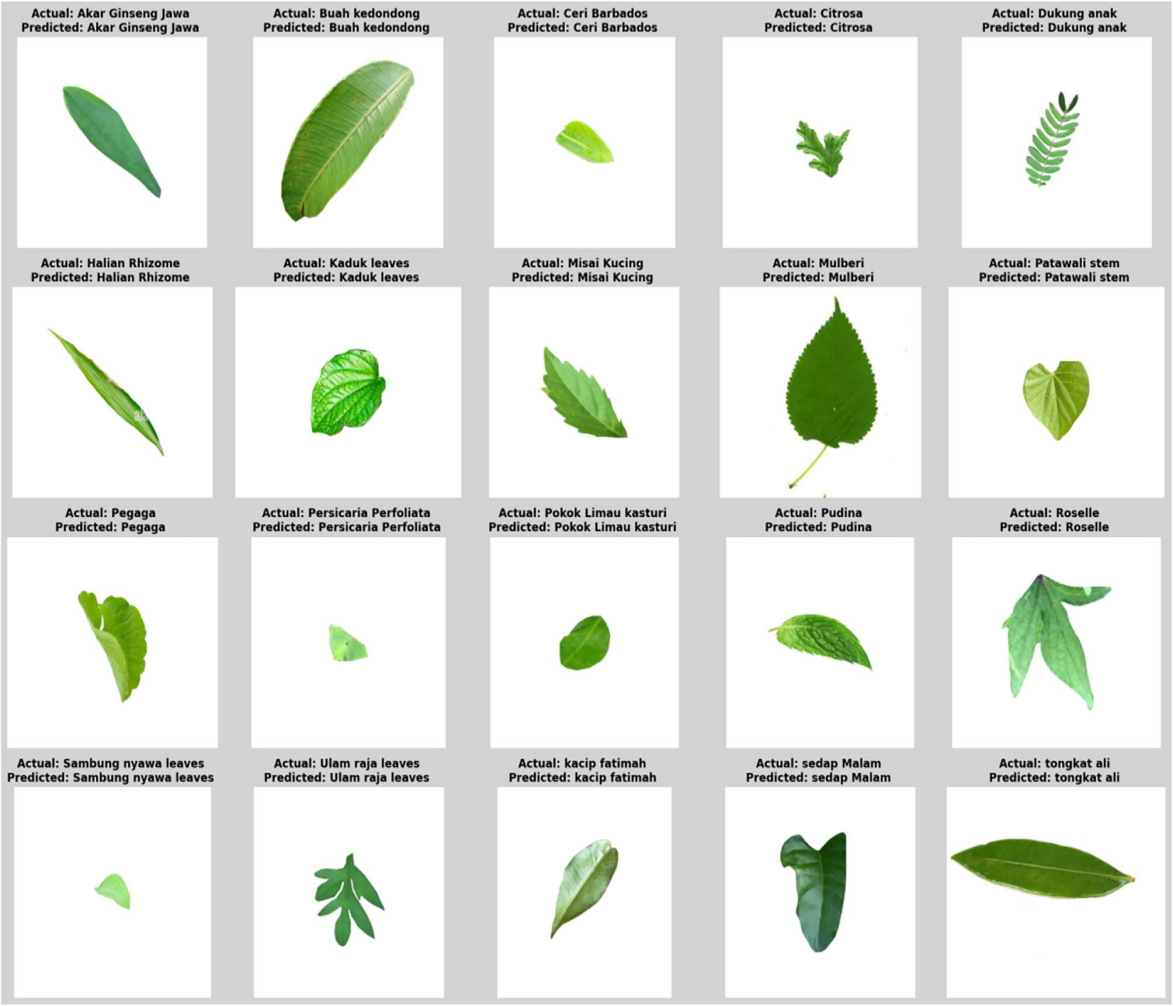
Actual vs. Predicted.

Figure 17 presents sample actual versus predicted results for different herbal leaf species from the FLAVIA dataset. For each example, the predicted label exactly matches the ground truth, demonstrating the method’s ability to accurately distinguish herbs with diverse shapes, sizes, and textures. The consistent agreement between actual and predicted classes highlights the strong feature representation and reliable classification performance of the proposed approach, even across visually varied herbal leaves.

#### Performance analysis of medical leaf dataset

**Fig. 18:**
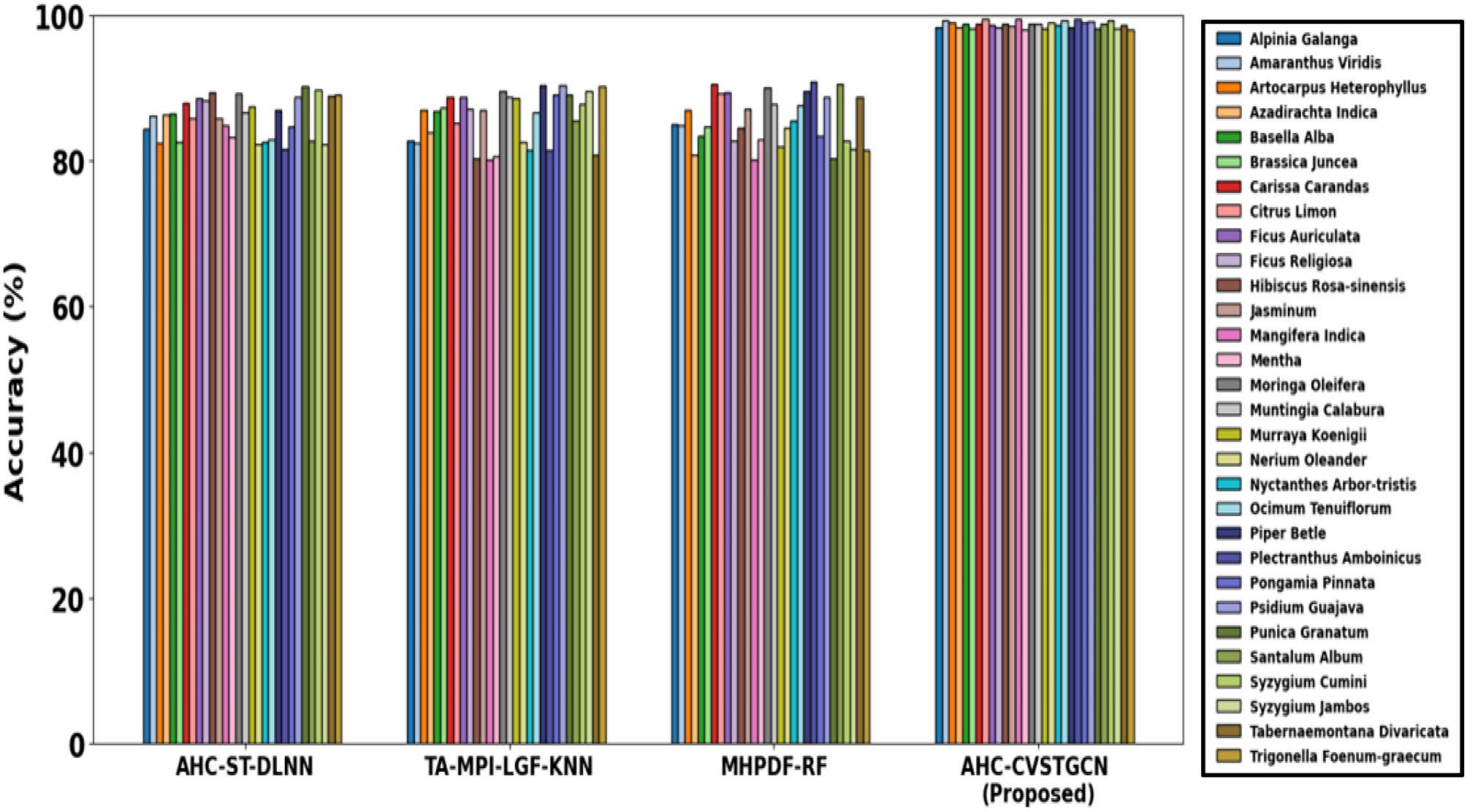
Analysis of accuracy of medical leaf dataset.

Figure 18 indicates the accuracy of the various models to classify the medical leaf dataset on 30 herbal species. Conventional models such as AHC-ST-DLNN, TA-MPI-LGF-KNN and MHPDF-RF are moderate in accuracy, with most of them being between 80 and 90 percent though there are other species that perform worse. On the contrary, the developed AHC-CVSTGCN model is always characterized by extremely high accuracy, which is approximately 98–99 percent with regards to all the species. This better performance can be attributed to the fact that the model can represent complex, spatial, and temporal relationships with the help of a complex-valued spatiotemporal graph network, optimized parameters with the help of HMRFO, noise reduction with the help of MLPF, as well as simultaneous extraction of shape, texture, and color features with the help of RQFWT that makes it more robust and discriminative than the existing methods.

**Fig. 19:**
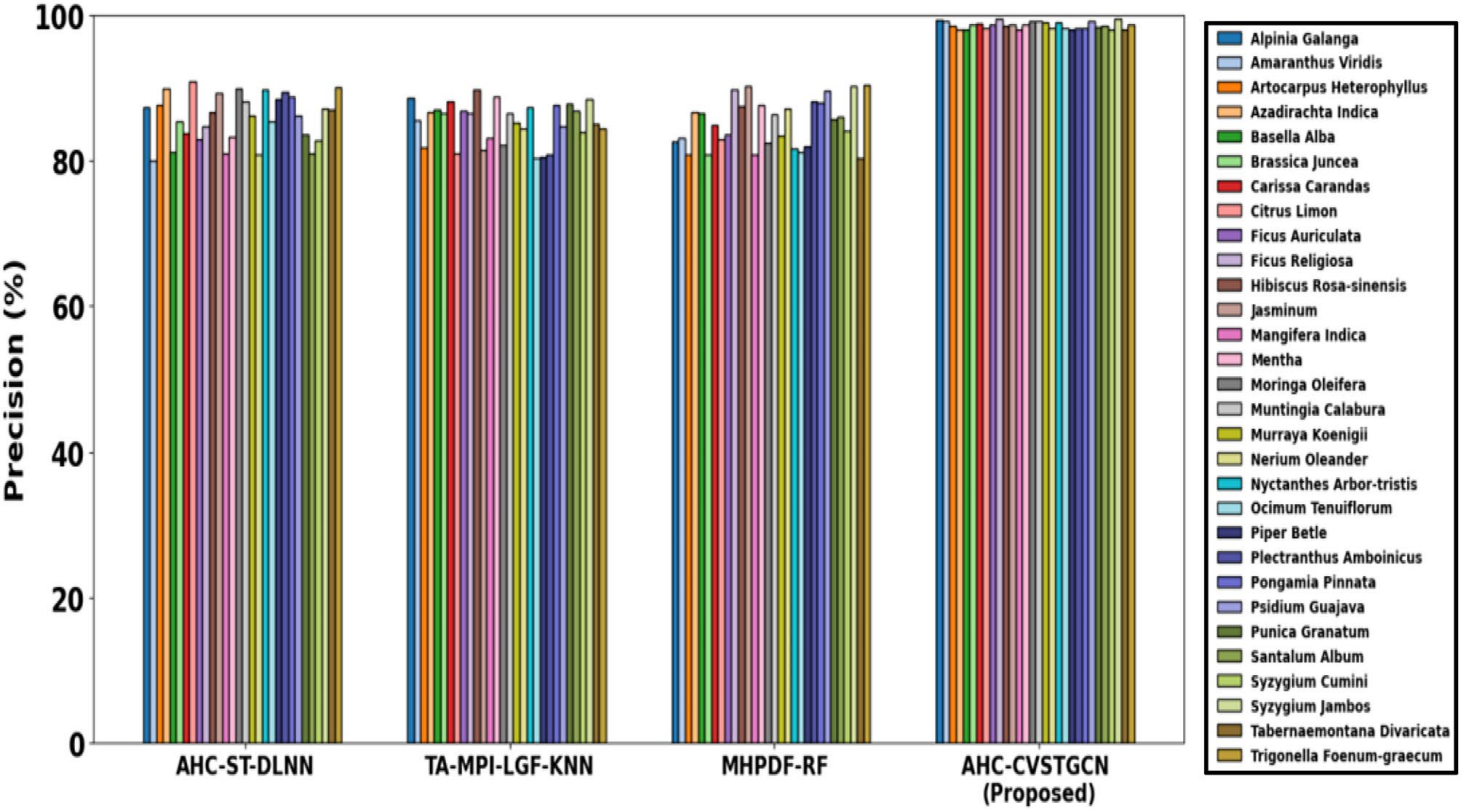
Precision analysis of medical leaf dataset.

Figure 19 demonstrates the accuracy of various models on the medical leaf data on various herbal species. The findings indicate that the proposed AHC-CVSTGCN algorithm attains the highest precision of nearly 99.9 almost on all species with the proposed baseline models as AHC-ST-DLNN, TA-MPI-LGF-KNN, and MHPDF-RF having a range of 80 and above. The outstanding results of AHC-CVSTGCN could be explained by the possibility to describe the complex spatial and temporal relationships in the leaf features with the complex-valued spatiotemporal graph structure, along with the effective feature extraction with the RQFWT and the background noise removal with the MLPF. This feature representation, which is comprehensive and represents the hierarchical parameter optimization provides more discriminative and accurate classification than the standard methods.

**Fig. 20:**
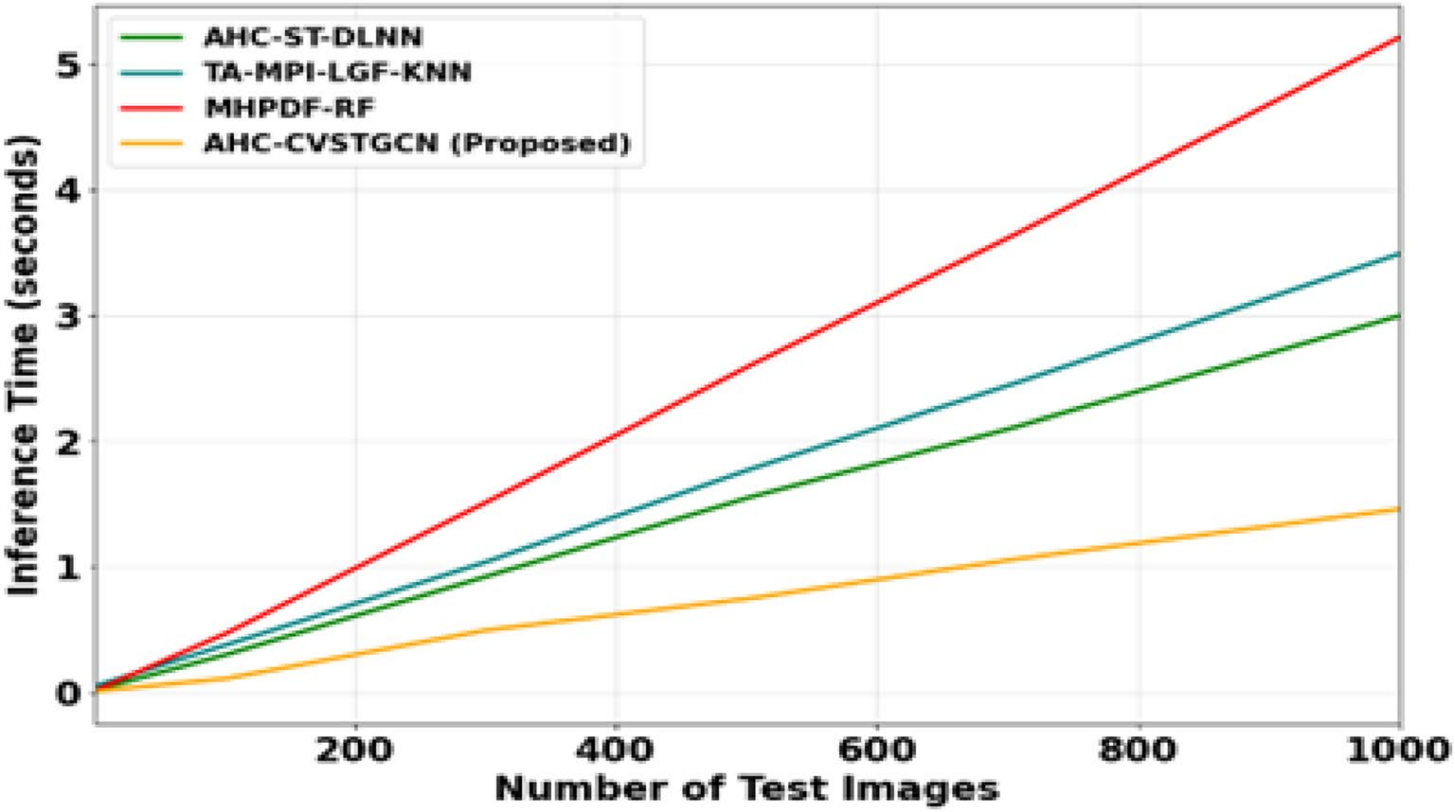
Performance analysis of computational scalability of medical leaf dataset.

Figure 20 shows the computational scalability of various models training on the medical leaf data by showing the inference time versus the number of test images. The proposed AHC-CVSTGCN has the highest performance with an inference time of about 1.5 s on 1000 test images compared to MHPDF-RF which has an inference time of more than 5 s, TA-MPI-LGF-KNN with an inference time of about 3.5 s and AHC-ST-DLNN with an inference time of about 3 s on 1000 test images. AHC-CVSTGCN is superiorly efficient because it has optimized graph based architecture and is hierarchically parameterized through HMRFO that minimizes computational overhead at high precision. This not only enables the proposed method to be highly accurate but it is also scalable and applicable to large-scale herbal classification tasks.

**Table 3:** Ablation study of AHC-CVSTGCN model configurations.

Ablation setup	Accuracy (%)	Precision (%)	Recall (%)
Proposed (AHC-CVSTGCN)	99.40	99.11	99.12
Without MLPF	97.85	96.42	97.10
Without HMRFO	96.02	96.80	97.35
Without MLPF & HMRFO	95.40	94.95	94.68

Table 3 presents the ablation study conducted to evaluate the contribution of different components within the proposed AHC-CVSTGCN model. The complete model achieves the highest performance with 99.40% accuracy, 99.11% precision, and 99.12% recall, demonstrating its effectiveness. When the MLPF module is removed, performance drops noticeably to 97.85% accuracy, indicating its importance in preprocessing and noise reduction. Similarly, excluding the HMRFO results in a further decrease, yielding 96.02% accuracy, this highlights the significance of optimization in enhancing classification. The performance declines even more when both MLPF and HMRFO are excluded, achieving only 95.40% accuracy, 94.95% precision, and 94.68% recall, confirming that both components play a crucial role in maximizing the model’s overall effectiveness.

Table 4 shows the statistical validation of the performance of the herb classification by comparing the proposed AHC-CVSTGCN model with the current methods in terms of accuracy and *p*-value analysis. The proposed AHC-CVSTGCN has the highest accuracy, which is 99.4, having surpassed AHC-ST-DLNN (91.2), TA-MPI-LGF-KNN (96.9), and MHPDF-RF (98.5). The performance improvements of the proposed method over these approaches are statistically significant and not the product of chance since all of the *p*-values are less than 0.001, which is much less than the significance level of 0.05. As AHC-CVSTGCN is the reference model, it does not have any *p*-values. In general, the findings support the strength and statistical excellence of the proposed method of classifying herbs.

**Table 4:** Statistical validation of herb classification performance.

Methods	Accuracy (%)	*P*-value (Proposed vs. Existing)	Significant ($P < 0.05$)
AHC-ST-DLNN	91.2	$< 0.001$	Yes
TA-MPI-LGF-KNN	96.9	$< 0.001$	Yes
MHPDF-RF	98.5	$< 0.001$	Yes
AHC-CVSTGCN (Proposed)	99.4	–	–

Table 5 displays how the performance of the AHC-CVSTGCN model is affected by different combinations of population size and learning rate during optimization. Smaller population sizes and lower learning rates lead to higher sensitivity, with the best result of 98.9% achieved using a population size of 30 and a learning rate of 0.001. In contrast, larger populations and higher learning rates reduce sensitivity, highlighting the importance of carefully tuning these hyperparameters to achieve more accurate and stable classification.

**Table 5:** Sensitivity analysis for varying population size and learning rate.

Population size	Learning rate	Sensitivity (%)
50	0.01	89.8
50	0.001	92.6
30	0.01	96.5
30	0.001	98.9

Table 6 demonstrates the generalization performance of the proposed AHC-CVSTGCN model across multiple datasets. While the highest accuracy, precision, and recall are achieved on the FLAVIA dataset (99.40%, 99.11%, and 99.12%), the model consistently maintains strong performance above 95% on other datasets despite variations in image quality, background, and leaf morphology. This confirms the robustness and cross-dataset reliability of the proposed approach for herbal species classification.

**Table 6:** Performance evaluation and generalization analysis of the AHC-CVSTGCN model across diverse botanical datasets.

Dataset	Accuracy	Precision	Recall
FLAVIA	99.40	99.11	99.12
Herb (Plant) classification	97.85	97.42	97.60
Phantom-fs/Herbify	96.92	96.35	96.48
Indian medicinal leaves	95.68	95.10	95.34

Table 7 is a comparison of the time required to execute the proposed AHC-CVSTGCN model with the available methods of classifying herbs. The proposed model has the shortest execution time of 15 min compared to Attention-based Enhanced Local and Global Features Network (AELGNet) which has 22 min Hierarchical Contextual Vision Integration Network (HCVINet) with 21 min, variable correlation kernel convolutional neural network (VCKCNN) and 19 min. The short execution time can be explained by the fact that its feature extraction with RQFWT and subsequent learning with HMRFO are efficient and allow processing at a much faster rate without any loss to the classification performance.

**Table 7:** Comparison execution time of proposed and existing methods.

Method	Execution time (mins)
AELGNet^34^	22
MFFN-CNN-HLCSO^35^	21
VCKCNN^36^	19
Proposed	15

Table 8 gives a comparative analysis between the proposed AHC-CVSTGCN model and existing transformer in terms of accuracy, efficiency, and inference time. The proposed model achieves the highest classification accuracy of 99.40%, outperforming ST, VT, and ConvNeXt. It also demonstrates strong efficiency of 98.6% while requiring the lowest inference time of 1.0 s, indicating faster prediction capability. In contrast, transformer-based models show lower accuracy and higher inference times, particularly Vision Transformer and ConvNeXt, which require substantially more time for inference. These results confirm that the proposed AHC-CVSTGCN offers a better balance between efficiency and accuracy while maintaining faster inference, thereby justifying its architectural design.

**Table 8:** Comparison of proposed and existing transformer.

Methods	Accuracy (%)	Efficiency (%)	Inference time (s)
Swin transformer (ST)	95.3	94.4	1.8
Vision transformer (VT)	91.7	90.8	2.4
ConvNeXt	88.67	97.5	4.7
Proposed	99.40	98.6	1.0

### Discussion

The discussion of the proposed AHC-CVSTGCN method shows that the performance of the model in automated herbal classification is always high and better than the current models when applied to a broad range of species. This excellent performance is supported by the synergistic combination of its main parts: MLPF preprocessing results in the removal of background noise and irrelevant features and preserves important herb characteristics; RQFWT feature extraction results in the ability to capture important shape, texture and color information with a high time-frequency resolution, allowing the model to discern subtle differences between similar species; CVSTGCN learning models capture spatial and temporal dependencies between features, its classification is more accurate and robust; and HMRFO optimization is used to fine-tune the model parameters, reducing misclassifications and maximizing sensitivity and specificity Ablation experiments have established that all the components are essential since removing any of them seriously impairs performance, and statistical tests have proven that better performance relative to current methods is strong and not by chance. Furthermore, the proposed AHC-CVSTGCN model can have a significant advantage in practical implementation and small-scale industries due to the high accuracy of the final results and reliability, in addition to automation of herb classification, a reduction in terms of time, cost-saving, and efficient sorting, verification, and quality-checking of raw herbs. Its rapid performance and strong workability across diverse environments render it applicable in the deployment to resource-limited environments, and it is capable of supporting scalable and evidence-based operations within the medicinal plant and agricultural industry. Overall, the advanced preprocessing, feature extraction, graph-based learning, and optimization make a combination of a very effective, generalizable, and practical solution to large-scale herbal classification.

## Conclusion

In conclusion, the proposed AHC-CVSTGCN model, integrating MLPF for noise reduction, RQFWT for robust feature extraction, CVSTGCN for advanced graph- based learning, and HMRFO for optimal parameter tuning, has proven to be a highly accurate and efficient solution for automated herbal classification. With an overall accuracy of 99.40%, precision of 99.11%, and recall of 99.1%, the model significantly outperforms existing approaches across multiple performance metrics. The proposed AHC-CVSTGCN technique achieves high classification accuracy on several herb species and datasets, and thus it has robustness and reliability. The RQFWT is very good at configuring the shape, texture, and color characteristics; whereas the MLPF preprocessing eliminates the noise in the background and minimizes misclassifications. The optimization of the network using HMRFO reduces false positives and negatives and enhances generalization. This highlights the effectiveness, robustness, and practical potential of the AHC-CVSTGCN model for reliable automated herbal classification across diverse datasets.

### Limitations and future work

Although the proposed AHC-CVSTGCN model demonstrates high accuracy and efficiency, certain limitations remain. Its performance may be affected when classifying unseen species or herbal leaves captured under highly variable field conditions, and its effectiveness in real-world deployment has not yet been fully validated. Future research will focus on expanding datasets to cover a broader range of species and variations, integrating spectral or chemical data to complement visual features for more robust classification and exploring real-time deployment of the model for practical applications in medicinal plant monitoring and processing. These improvements aim to further increase the model’s robustness and practical applicability in large-scale herbal classification tasks.

## Data Availability

The FLAVIA dataset was obtained from Kaggle and was collected from https://www.kaggle.com/datasets/gauravneupane/flavia-dataset. The Medical leaf dataset was collected from https://www.kaggle.com/datasets/anurag629/medical-leaf-dataset.

## Abbreviations

AHC: Automated herb classification
MLPF: Multiple local particle filters
CVSTGCN: Complex-valued spatio temporal
CNN: Convolutional neural network
PSO: Particle swarm optimization
VT: Vision transformer
KNN: K-nearest neighbour
VT: Vision transformer
LSTM: Long short term memory
TPR: True positive rate
HCVINet: Hierarchical contextual vision integration network
VCKCNN: Variable correlation kernel convolutional neural network
SVM: Support vector machine
RQFWT: Revised tunable Q-factor wavelet transform
HMRFO: Hierarchical manta ray foraging optimization
DLNN: Deep learning neural network
RF: Random forest
MLP: Multi-layer perceptron
DCNN: Deep convolutional neural networks
MLP: Multi-layer perceptron
FNR: False-negative rate
ST: Swin transformer
RQFWT: Revised tunable Q-factor wavelet transform
AELGNet: Attention-based enhanced local and global features network

## List of symbols

*g*_*b*_: Updating the population
*y*_*l*_*, x*_1−__*l*_: Local region identification
*x*^*i*^: Outliers
*h*^*i*^: The particle generation
*y*^*i*^ and *y*^(^^*i,j*^^)^: Inaccurate data
*θ*(·): Operations used to detect and minimize measurement errors
*h*_*i*_: Subspace in filter
*α*: Image reconstruction
*P*: Bandwidth has a zero point
*d*_*R*_: Designed to meet the criteria
*R*^*t*^: Sub-band
*j*: Sampling point
$\left| {\tilde{B}} \right|$: Cardinal number of the set
$\tilde{B}_{j}$: Common characteristic subspaces
*D*: Define the colour feature
*G*(*R*): The linear matrix operator
*J*: The length of the graph
*G*_*s*−*τ*_ (*R*): Linear matrix operator
*g*_*j*__,__*s*_: Real scalar value in times
*y*: The transform
*W*: Multiple channel
$$H(\Lambda ,y)$$: Diagonal matrix
$\overline{v}$: Element multiplication of the graph filter
*g*_*j*_: Real scalar value
*C*: Matrix of prime populations
*v*_*s*_: Maximizing the accuracy
*B*_*j*_: Gap between the greatest person
*P*_*j*_: The *i*th individual’s score
*E*_*j*_: The fitness value
*y*^*b*^: Updating the population
*w*: Most iterations possible
*y*^*j*^: Various features of the image
*x*_1−__*l*_: Neighbour data in filter layer
*e*: Random variable
*v*^(*i*^^,^^*j*^^)^: Data flows
*y*_0:__*l*__−2_ and *y*_*i*_: Weighted particle fusion
$$\tilde{v}^{{\left( {i,j} \right)}}$$: Removed outliers
*∂ω*^*j*^: Transform function
*i*: Center frequency
*π*: Data function
*a*^*t*^: Sub band coefficient
*M*^*T*^: Bandwidth of the filter
*j*: Represents the sampling point
*r*^*t*^: Error function
*s*(*r*, *g*, *b*): Functions as a map between the red
*R*: Matrix operator
*g*_*j*_: The real scalar value
*w*_*τ*_: Graph point
*V*: Multiple channel output
*G*(*R*): Linear matrix operator
⊗: Tensor product
*g*_*j*__−__*s*_: Real scalar value
*σ*(·): Input complex values
*R*: Matrix operator
*E*: *i*^*th*^ individual’s objective function value
$\overline{v}$: Increasing the ROC
*i*^*th*^: Individual
*δ*: Functional weights
*B*_*j*_: The distance between the *i*th individual
*v*: The most iterations possible
